# Yield and Nutritional Characterization of Thirteen Quinoa (*Chenopodium quinoa* Willd.) Varieties Grown in North-West Europe—Part I

**DOI:** 10.3390/plants10122689

**Published:** 2021-12-07

**Authors:** Phara De Bock, Filip Van Bockstaele, Hilde Muylle, Paul Quataert, Pieter Vermeir, Mia Eeckhout, Gerda Cnops

**Affiliations:** 1 Research Unit of Cereal and Feed Technology, Department of Food Technology, Safety and Health, Faculty of Bioscience Engineering, Ghent University, 9000 Ghent, Belgium; mia.eeckhout@ugent.be; 2 Food Structure and Function Research Group (FSF), Department of Food Technology, Safety and Health, Faculty of Bioscience Engineering, Ghent University, 9000 Ghent, Belgium; filip.vanbockstaele@ugent.be; 3 Plant Sciences Unit, Flanders Research Institute for Agriculture, Fisheries and Food (ILVO), 9090 Melle, Belgium; hilde.muylle@ilvo.vlaanderen.be (H.M.); paul.quataert@ilvo.vlaanderen.be (P.Q.); gerda.cnops@ilvo.vlaanderen.be (G.C.); 4 Laboratory for Chemical Analysis (LCA), Department of Green Chemistry and Technology, Faculty of Bioscience Engineering, Ghent University, 9000 Ghent, Belgium; pieter.vermeir@ugent.be

**Keywords:** *Chenopodium quinoa* Willd., North-West Europe, yield, saponins, amino acids, fatty acids, triacylglycerols, minerals

## Abstract

The cultivation of quinoa has gained increasing interest in Europe. Different European varieties exist, but more research is required to understand the individual variety characteristics for end-use applications. The objective of this study is to evaluate the agronomic performance of 13 quinoa varieties under North-West European field conditions during three growing seasons (2017–2019). Furthermore, seeds were qualitatively characterized based on characteristics and composition. Yield differed among varieties and growing seasons (0.47–3.42 ton/ha), with lower yields obtained for late-maturing varieties. The saponin content varied from sweet to very bitter. The seeds contained high protein levels (12.1–18.8 g/100 g dry matter), whereas varieties had a similar essential amino acid profile. The main fatty acids were linoleic (53.0–59.8%), α-linolenic (4.7–8.2%), and oleic acid (15.5–22.7%), indicating a high degree of unsaturation. The clustering of varieties/years revealed subtle differences between growing seasons but also reflected the significant interaction effects of variety and year. Most varieties perform well under North-West European conditions, and their nutritional content is well within the values previously described for other cultivation areas. However, optimal yield and quality traits were not combined in one variety, illustrating the importance of breeding for adapted quinoa varieties.

## 1. Introduction

Quinoa (*Chenopodium quinoa* Willd.) is a highly nutritional and resilient crop, native to the Andean region. It is a pseudocereal belonging to the *Amaranthaceae* family that was domesticated and cultivated more than 5000 years ago [1,2]. Unlike the other staple crops of the Andes, quinoa remained relatively unknown outside the Andean region until it was exported as a food product to the USA in the late 1970s. From that period onwards, quinoa consumption increased worldwide [2,3]. As the high nutritional value of quinoa seeds started to be recognized, this pseudocereal gained increasing global interest in the last decade [1,4]. The quinoa seeds can be used for the same purpose as cereals, such as wheat or rice, or as whole grains in salads, cooked meals, breakfast, or soups. They can be milled into flour to produce pasta, bread, biscuits, and pancakes. The seeds can be used for the production of dairy-free drinks or, in fermented form, for the production of beer or the traditional *chicha* drink [5,6].

The start of quinoa exports and consumer interest outside the Andean region rapidly increased the demand and price of quinoa in the main producing countries, Bolivia and Peru [2,4]. The subsequent expansion of the agricultural frontier and the unsustainable practices in South America emphasized the need to grow quinoa in other parts of the world [2,7]. In the 1980s, breeding programs were initiated in several European countries, i.e., the UK, Denmark, and the Netherlands. The research focused on the adaptation to European climatic conditions and the improvement of agronomic performance (e.g., early maturity, high yield, low saponin concentration, no head sprouting). Chilean varieties belonging to the coastal ecotype are less sensitive to photoperiods, an important requisite for northern latitudes, and were the ideal starting material for European varieties [3,4,8]. Nowadays, several Dutch and Danish varieties have been registered in Europe. The newest varieties show early maturity and are almost completely day-length neutral [3,9]. Moreover, Patiranage et al. [10] recently identified haplotypes that induce photoperiod insensitivity associated with early flowering under long day lengths. A haplotype-based breeding strategy of pyramiding favorable alleles is suggested by the authors to breed quinoa varieties that are early maturing under long day lengths and are, thus, suitable for cultivation in northern latitudes [10].

As a pseudocereal, quinoa has distinctive features from conventional cereals. Its protein content is substantially higher (7–23%) than that of the staple crops rice, barley, corn, and rye and slightly higher than that of wheat, with a better balanced essential amino acid profile [11,12]. Moreover, quinoa has a higher lipid content with a good representation of essential fatty acids, a higher ash content, and higher concentrations of vitamins E, C, B2, B6, and folic acid. Besides antioxidants, quinoa contains several other phytochemicals with positive effects on human health and nutrition. Furthermore, quinoa is generally safe to eat for patients with celiac disease [2,4,11,12,13].

Quinoa is characterized by a broad genetic diversity, which allows adaptability to diverse climates and cultivation in a wide range of environments such as highlands, coastal regions, subtropical environments, or arid regions. Furthermore, its ability to grow under various abiotic stress conditions (i.e., frost, salinity, drought) makes quinoa a promising crop for further expansion in many parts of the world to help feed the world’s population in the context of a changing climate and food security [8,14,15,16]. 

Seed quality is a complex trait that results from the interaction between genetic and environmental factors, and, except for saponin content, it has gotten little attention in breeding programs [4,15]. There is also little information available on the nutritional quality of the different European varieties when produced in Europe, compared to quinoa produced in South America [4,15,17]. Insufficient knowledge of the characteristics and their consequences for potential end-uses complicate the use of European quinoa in the food industry. Therefore, more research is required to gain a better understanding of the individual variety characteristics for end-use applications and to bridge the gap between farmers and end-users [16]. The objective of this study is to perform comparative variety testing with 10 European bred varieties and 3 Farm Original varieties over 3 consecutive growing seasons (2017–2019) [18] to (i) evaluate their agronomic performance under North-West European field conditions and to (ii) qualitatively characterize the seed based on characteristics and composition. In addition, principal components analysis (PCA) and hierarchical cluster analysis (HCA) were employed to group the varieties/years into clusters with similar yield, characteristics, and macronutrient composition.

## 2. Results and Discussion

### 2.1. Yield

**Figure 1:**
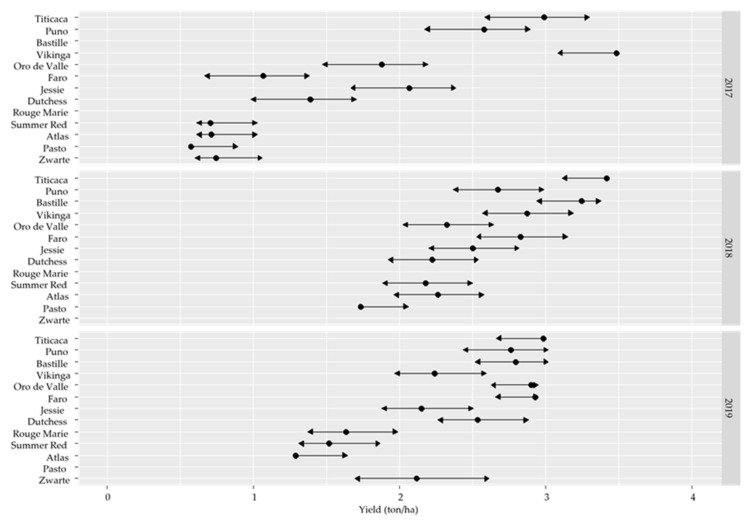
Yield (ton/ha) of 13 quinoa varieties grown under North-West European field conditions in 2017, 2018, and 2019 (*n* = 3).

The overall mean and range of the yield (Figure 1) of the different varieties was strongly year-dependent, with the widest spread in 2017 (0.57–3.48 ton/ha). The ranking of the varieties was, however, mostly consistent over the tested growing seasons. Titicaca, Bastille, Vikinga, and Dutchess were the best performing varieties under the North-West European weather conditions in 2017–2019. The three dark-colored (i.e., Rouge Marie, Summer Red, and Zwarte) varieties were less yielding compared to most white varieties and similar to Atlas and Pasto, with consistently lower yields. Moreover, the varieties clearly differed in the length of their growing season, which ranged between 111 and 187 growing days (GDs). Jessie, Vikinga, and Titicaca belonged to the early varieties, and Atlas and Pasto were consistently the latest to be harvested. The seed yields were comparable to the yields reported in other field trials [4,19]. In two previous studies, a small subset of the analyzed varieties was grown under European conditions, i.e., Puno, Titicaca, and Jessie in Germany [4] and Vikinga, Titicaca, and Puno in Spain [19]. The present seed yield data were in the range of the Spanish study (0.70–3.25 ton/ha, [19]) and higher than the German study (1.73–2.43 ton/ha, [4]). However, the same varieties needed similar GDs as in Germany [4] or Spain [19].

A significant negative correlation (*r* = −0.705, *p* < 0.001) was found between yield and GDs (Figure S1) but was probably biased by the varieties Atlas and Pasto. These late varieties were not able to reach full maturity under the North-West European growth conditions before 15 September. Results from the EU project Protein2Food suggested that yield potential in quinoa can be influenced by the sowing date [20]. In the 2017–2019 trials, the sowing date was the same for early, mid, and late varieties and depended on the local field and weather conditions. An earlier sowing date for mid-late and, especially, late varieties might be beneficial and, in some years, even required for completing their life cycle in favorable weather conditions [20]. Earlier sowing dates (before 15 April), as recommended by Radicle Crops for their varieties (pers. comm.), might have resulted in higher yields, mainly for the late varieties (i.e., Pasto and Atlas) in 2018 and 2019. The European-bred varieties, except for the late varieties (i.e., Atlas and Pasto), did perform well under the North-West European growth conditions. The Farm Original varieties (i.e., Faro, Oro de Valle, and Zwarte) were taller, phenotypically less homogenous (data not shown), and mid-late maturing and, except for Zwarte, performed similarly to most European-bred varieties.

### 2.2. Seed Size and Weight

Seed size, thousand seed weight (TSW), and test weight differed significantly among varieties and years, with significant variety × year interactions. The quinoa seeds had a length of 1.65 to 2.25 mm and a width of 1.50 to 1.99 mm (Table 1), which corresponded to a length–width ratio (LWR) of 1.09 to 1.14 (Table S1). This is within the range of seed sizes reported by Bhargava et al. (1.34–2.21 mm, [17]) and Bertero et al. (1.8–2.2 mm, [21]). The ranking of the varieties according to seed size was inconsistent over the years. For example, the seeds of Puno and Vikinga were the smallest in 2019, while the growing season of 2018 resulted in the smallest seeds for Oro de Valle, Bastille, and Dutchess. The seed size of Faro, Jessie, and Summer Red remained stable in 2018 and 2019, while Pasto had a stable seed size in 2017 and 2018. In general, the growing season of 2017 resulted in the largest seeds. Among varieties, Puno and Pasto were characterized by the smallest seed size. Zwarte and Summer Red generally had the largest seeds. Over the years, Atlas and Jessie had comparable seed sizes. 

The TSW of the quinoa varieties (Table 1) ranged between 1.90 and 3.68 g. These values corresponded with the ranges reported for studies in Germany (1.2–3.3 g, [4]), Italy (1.77–3.63 g, [22]), and Spain (1.7–3.4 g, [19]), in which four varieties were common to this study. Most varieties obtained the lowest TSW in 2018. This growing season was characterized by a prolonged dry period with 79% less overall rain compared to 2017 (Figure 5). Insufficient rain can reduce TSW during the seed filling phase [4]. However, the growth conditions in 2018 resulted in the largest seeds with the highest TSW for Titicaca. The TSW of Atlas remained stable at around 2.33 g in 2018 and 2019, while other varieties had a higher TSW in 2019. Overall, the highest TSW was obtained in 2017, except for Faro, Oro de Valle, and Titicaca. The two Farm Original varieties (i.e., Faro and Oro de Valle) reached the highest TSW under the growth conditions of 2019. Across growing seasons, the dark-colored varieties (i.e., Summer Red, Rouge Marie, and Zwarte) produced the largest seeds with the highest TSW, while the Puno seeds were the smallest and the lightest. The ranking of Puno, Jessie, and Titicaca (from small to high) was identical to the ranking by Präger et al. [4] in Germany. The present data support the influence of the genetic factor determining TSW. There is still room for increasing seed size and TSW through breeding using varieties from the coastal ecotype or incorporating this trait from other quinoa ecotypes, e.g., the *salares* ecotype (the real quinoa varieties from Bolivia), of which the seeds have the highest TSW [23,24]. 

The test weight (Table 1) varied between 68.9 and 82.5 kg/hL, which is higher compared to the test weight of quinoa grown in Bolivia (66.3–73.0 kg/hL, [25]) or the USA (66.1–75.7 kg/hL, [25]). In 2019, the test weight of the quinoa seeds was significantly lower compared to other growing seasons. However, Pasto and Faro had the lowest test weight in 2017. The highest test weights were measured in 2018 for all varieties, except for Atlas and Titicaca. The latter reached the highest test weight under the growth conditions of 2017. The test weight of Puno and Titicaca was usually high, especially in 2017 and 2018. In 2019, the highest test weights were measured for Zwarte and Faro. Wu et al. [25] concluded that Oro de Valle seeds had a higher test weight than Black seeds (possible origin for variety Zwarte). Within the present study, Zwarte seeds invariably had a higher test weight compared to Oro de Valle seeds. Despite the comparable seed size, Atlas and Jessie had different test weights in 2017 and 2018. These varieties also significantly differed in TSW, which suggests differences in seed composition. Dutchess invariably had the lowest test weight in comparison to the different quinoa varieties.

**Table 1:** Seed characteristics and saponin content (mg/g) of 13 quinoa varieties grown under North-West European field conditions in 2017, 2018, and 2019 (*n* = 3).

Year	Variety	Length (mm) ^1^	Width (mm) ^1^	TSW (g) ^1,2^	Test Weight (kg/hL) ^1^	Saponins (mg/g) ^1,2^
2017	Atlas	1.97 ± 0.00 ^d,B^	1.80 ± 0.00 ^f,B^	2.85 ± 0.01 ^e,B^	75.9 ± 0.1 ^e,C^	<LoQ
	Bastille	-	-	-	-	-
	Dutchess	1.96 ± 0.00 ^d,B^	1.80 ± 0.01 ^f,B^	2.94 ± 0.01 ^f,g,C^	71.9 ± 0.2 ^a,B^	0.1 ± 0.0 ^a,A^
	Faro	1.86 ± 0.01 ^c,B^	1.69 ± 0.00 ^c,B^	2.56 ± 0.01 ^c,B^	74.9 ± 0.2 ^c,d,A^	1.7 ± 0.6 ^c,d,A^
	Jessie	1.95 ± 0.01 ^d,B^	1.79 ± 0.01 ^e,f,C^	2.76 ± 0.02 ^d,C^	75.3 ± 0.2 ^d,B^	<LoQ
	Oro de Valle	1.97 ± 0.01 ^d,C^	1.76 ± 0.01 ^e,C^	2.98 ± 0.01 ^g,B^	74.6 ± 0.1 ^b,c,B^	1.1 ± 0.2 ^b,c,A^
	Pasto	1.77 ± 0.00 ^b,A^	1.61 ± 0.01 ^b,A^	2.37 ± 0.01 ^b,B^	74.7 ± 0.2 ^b,c,A^	< LoQ
	Puno	1.72 ± 0.01 ^a,C^	1.56 ± 0.01 ^a,B^	2.27 ± 0.01 ^a,C^	81.7 ± 0.2 ^g,B^	2.2 ± 0.2 ^d,A^
	Rouge Marie	-	-	-	-	-
	Summer Red	2.24 ± 0.01 ^e,B^	1.99 ± 0.00 ^g,B^	3.47 ± 0.02 ^h,C^	74.5 ± 0.1 ^b,B^	<LoQ
	Titicaca	1.88 ± 0.01 ^c,A^	1.69 ± 0.01 ^c,A^	2.87 ± 0.03 ^e,f,B^	82.5 ± 0.1 ^h,C^	0.4 ± 0.1 ^a,b,A^
	Vikinga	1.89 ± 0.00 ^c,C^	1.72 ± 0.00 ^d,C^	2.70 ± 0.01 ^d,C^	77.2 ± 0.1 ^f,B^	< LoQ
	Zwarte	2.25 ± 0.02 ^e,B^	1.98 ± 0.02 ^g,A^	3.68 ± 0.02 ^I,B^	77.6 ± 0.2 ^f,B^	0.8 ± 0.2 ^a,b,c,A^
2018	Atlas	1.89 ± 0.01 ^d,A^	1.74 ± 0.01 ^d,A^	2.32 ± 0.02 ^c,d,A^	75.6 ± 0.1 ^a,B^	<LoQ
	Bastille	1.87 ± 0.00 ^c,d,A^	1.68 ± 0.01 ^c,A^	2.49 ± 0.02 ^f,A^	78.1 ± 0.3 ^d,B^	<LoQ
	Dutchess	1.86 ± 0.01 ^c,A^	1.69 ± 0.01 ^c,A^	2.38 ± 0.04 ^d,e,A^	75.3 ± 0.2 ^a,C^	<LoQ
	Faro	1.80 ± 0.01 ^b,A^	1.64 ± 0.00 ^b,A^	2.28 ± 0.03 ^c,A^	79.6 ± 0.1 ^e,C^	5.2 ± 0.6 ^c,B^
	Jessie	1.90 ± 0.02 ^d,A^	1.75 ± 0.02 ^d,B^	2.24 ± 0.02 ^c,A^	76.8 ± 0.1 ^b,C^	<LoQ
	Oro de Valle	1.85 ± 0.00 ^c,A^	1.67 ± 0.01 ^c,A^	2.46 ± 0.01 ^f,A^	77.1 ± 0.1 ^b,C^	2.8 ± 0.9 ^b,B^
	Pasto	1.78 ± 0.01 ^b,A^	1.62 ± 0.01 ^b,A^	2.00 ± 0.03 ^b,A^	76.7 ± 0.1 ^b,B^	<LoQ
	Puno	1.68 ± 0.01 ^a,B^	1.54 ± 0.01 ^a,B^	1.90 ± 0.03 ^a,A^	81.6 ± 0.1 ^f,B^	5.1 ± 1.2 ^c,B^
	Rouge Marie	-	-	-	-	-
	Summer Red	2.05 ± 0.01 ^e,A^	1.88 ± 0.01 ^e,A^	2.95 ± 0.04 ^g,A^	78.3 ± 0.2 ^d,C^	<LoQ
	Titicaca	2.03 ± 0.02 ^e,B^	1.85 ± 0.02 ^e,C^	2.93 ± 0.04 ^g,C^	79.9 ± 0.1 ^e,B^	1.3 ± 0.4 ^a,B^
	Vikinga	1.85 ± 0.02 ^c,B^	1.68 ± 0.02 ^c,B^	2.43 ± 0.01 ^e,f,A^	77.6 ± 0.2 ^c,C^	<LoQ
	Zwarte	-	-	-	-	-
2019	Atlas	1.89 ± 0.01 ^c,A^	1.73 ± 0.00 ^c,d,A^	2.33 ± 0.02 ^b,A^	73.6 ± 0.2 ^c,d,A^	<LoQ
	Bastille	1.97 ± 0.01 ^d,B^	1.76 ± 0.01 ^d,B^	2.90 ± 0.06 ^e,B^	71.9 ± 0.2 ^b,A^	<LoQ
	Dutchess	2.00 ± 0.01 ^d,C^	1.83 ± 0.01 ^e,C^	2.77 ± 0.04 ^d,B^	68.9 ± 0.1 ^a,A^	<LoQ
	Faro	1.79 ± 0.01 ^b,A^	1.64 ± 0.01 ^b,A^	2.75 ± 0.02 ^d,C^	75.5 ± 0.3 ^g,B^	7.9 ± 0.1 ^e,C^
	Jessie	1.89 ± 0.01 ^c,A^	1.72 ± 0.00 ^c,A^	2.48 ± 0.02 ^c,B^	73.9 ± 0.2 ^d,e,A^	< LoQ
	Oro de Valle	1.89 ± 0.01 ^c,B^	1.73 ± 0.00 ^c,B^	3.04 ± 0.02 ^f,C^	73.4 ± 0.2 ^c,A^	5.4 ± 0.4 ^c,d,C^
	Pasto	-	-	-	-	-
	Puno	1.65 ± 0.01 ^a,A^	1.50 ± 0.01 ^a,A^	2.15 ± 0.03 ^a,B^	74.6 ± 0.3 ^f,A^	6.3 ± 0.5 ^d,C^
	Rouge Marie	1.98 ± 0.01 ^d,A^	1.83 ± 0.01 ^e,A^	3.27 ± 0.05 ^g,h,A^	73.5 ± 0.3 ^c,d,A^	<LoQ
	Summer Red	2.05 ± 0.02 ^e,A^	1.88 ± 0.01 ^f,A^	3.23 ± 0.02 ^g,B^	73.5 ± 0.2 ^c,d,A^	<LoQ
	Titicaca	1.88 ± 0.01 ^c,A^	1.71 ± 0.01 ^c,B^	2.79 ± 0.01 ^d,A^	74.5 ± 0.2 ^f,A^	3.2 ± 0.4 ^b,C^
	Vikinga	1.78 ± 0.01 ^b,C^	1.61 ± 0.01 ^b,A^	2.55 ± 0.03 ^c,B^	74.2 ± 0.2 ^e,f,A^	0.9 ± 0.4 ^a,A^
	Zwarte	2.19 ± 0.01 ^f,A^	1.99 ± 0.01 ^g,A^	3.35 ± 0.05 ^h,A^	75.9 ± 0.1 ^g,A^	4.8 ± 0.4 ^c,B^

^1^ Within years, average values followed by the same lowercase letter are not significantly different (*p* > 0.05). Capital letters compare the three years for the same variety; average values followed by the same letter are not significantly different (*p* > 0.05). ^2^ TSW: thousand seed weight, LoQ: limit of quantification (= 0.1 mg/g, [26]).

### 2.3. Seed Color

The seed color (L*a*b*) of the quinoa varieties is presented in Table S1. The variety × year interaction had a significant impact on the L*, a*, and b* values of the seed color. The luminosity (L*) of the white varieties ranged from 60.15 to 67.69 and was considerably higher than that of the dark-colored varieties (40.81–47.12). In 2017 and 2018, Pasto was characterized by the lowest luminosity among the white varieties, and Faro and Bastille by the highest. Moreover, Bastille had the clearest seeds in 2019. Lighter-colored seeds are generally preferred within white-seeded varieties [27]. The growing conditions in 2019 resulted in a higher luminosity for the dark-colored varieties. Among these varieties, Zwarte was characterized by the darkest seeds.

The red component of seed color was generally higher in 2019, although Faro seeds obtained the highest a* value in 2017. Rouge Marie had the reddest seeds (a* = 6.56) among all varieties, while Zwarte seeds were characterized by the lowest a* value (1.91). The a* value of the other varieties ranged between 3.72 and 5.42. The red component of Atlas and Puno seeds was generally lower compared to that of other white seeds. All varieties, except for Pasto and Vikinga, had comparable a* values in 2018. 

The b* value was considerably lower for dark-colored seeds and varied between 0.69 and 5.48. Besides a lower a* value, Zwarte seeds were also characterized by a lower b* value than Rouge Marie or Summer Red. Zwarte is a very heterogeneous variety with a segregation for seed color that is genetically determined by two genes [28]. This results in a mixture of red, brown, and black seeds with low a* and b* values. The b* value ranged from 15.23 to 20.72 for the white varieties, with the highest values measured in 2019. Only Vikinga seeds had a higher b* value in 2017. On average, Oro de Valle, Faro, Jessie, and Bastille were characterized by the highest yellow pigment.

Granado-Rodriguez et al. [19] studied the color of Puno, Titicaca, and Vikinga seeds over three years of cultivation in Spain. These authors reported lower L* values (50.3–63.1) and higher b* values (19.6–24.2). Sobota et al. [29] described the color of Faro, Puno, and Titicaca seeds cultivated in Poland. The results were within the ranges reported within the present study.

### 2.4. Saponins

The saponin content of the quinoa varieties is listed in Table 1. Depending on the saponin content, quinoa is classified as sweet or bitter. Quinoa can be considered sweet when its saponin content is 1.1 mg/g or less [26]. Atlas, Bastille, Dutchess, Jessie, Pasto, Rouge Marie, and Summer Red produced sweet seeds in all growing seasons. The saponin content of these varieties, except for Dutchess in 2017 (0.1 mg/g), was too low in all years to quantify. Saponins have an undesired bitter taste, and the bitter components decrease the nutritional value of the seed [12]. In contrast to other seed characteristics, saponin concentration has a high heritability, which makes it an important breeding trait [15,30]. The WUR and AbbottAgra varieties were bred for sweetness, and the saponin concentrations are extremely low due to a genetic mutation in the T-SARL1 transcription factor [31]. The saponin concentration of the other varieties ranged from 0.1 to 7.9 mg/g, with Faro as the bitterest variety. Vikinga is another variety bred for sweetness. The present data confirmed this, but a higher saponin content of 0.9 mg/g was observed for Vikinga seeds from 2019. Granado-Rodríguez et al. [19] found an overall higher saponin content for Vikinga, with the highest content in 2019 (2.04 g/100 g) as well. This, together with the present data, suggests genetic segregation or cross-contamination rather than environmental conditions, especially because the Vikinga plots were less homogenous for other traits, such as plant height, stem color, and maturing in 2019 (data not shown). The lowest saponin concentrations were observed in 2017, whereas varieties classified as bitter had remarkably lower saponin content in 2018 and 2019. Oro de Valle, Titicaca, and Zwarte (0.4–1.1 mg/g) were even considered sweet in 2017. The highest saponin concentrations were measured in 2019, with ranges for the bitter varieties between 3.2 and 7.9 mg/g. Compared to varieties also analyzed by Präger et al. (0.0–3.4 mg/g, [4]), the saponin concentrations in the present study were in the same line for Jessie and Titicaca but higher for Puno (2018 and 2019). Medina-Meza et al. [32] qualified Oro de Valle and Black (possible origin for variety Zwarte) as low saponin content varieties and Puno and Titicaca as bitter varieties. This is in accordance with the present study, except for the saponin content of Titicaca seeds from 2017.

### 2.5. Chemical Composition

#### 2.5.1. Macronutrients

**Figure 2:**
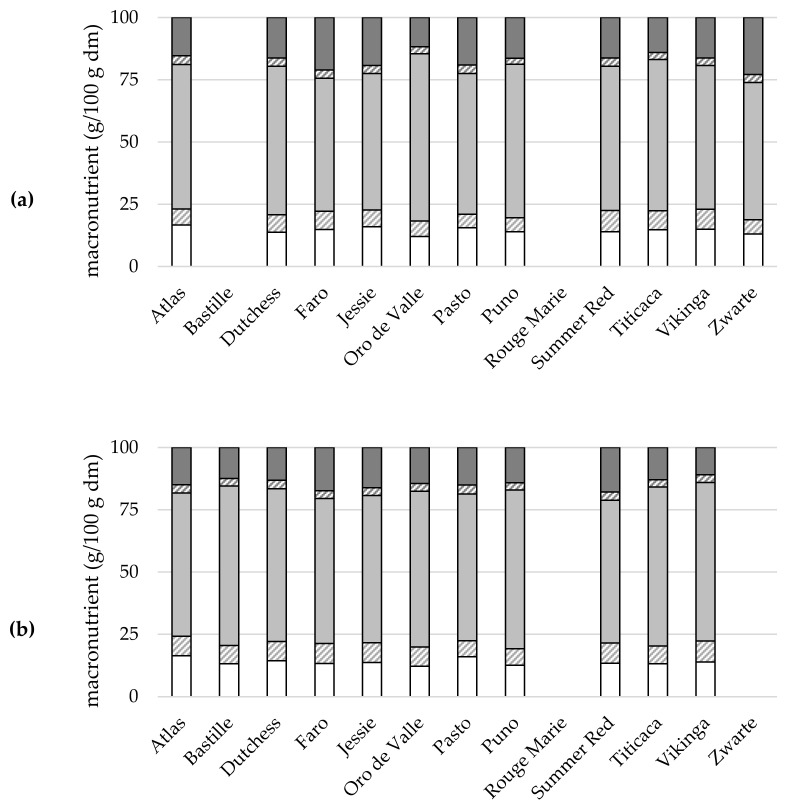

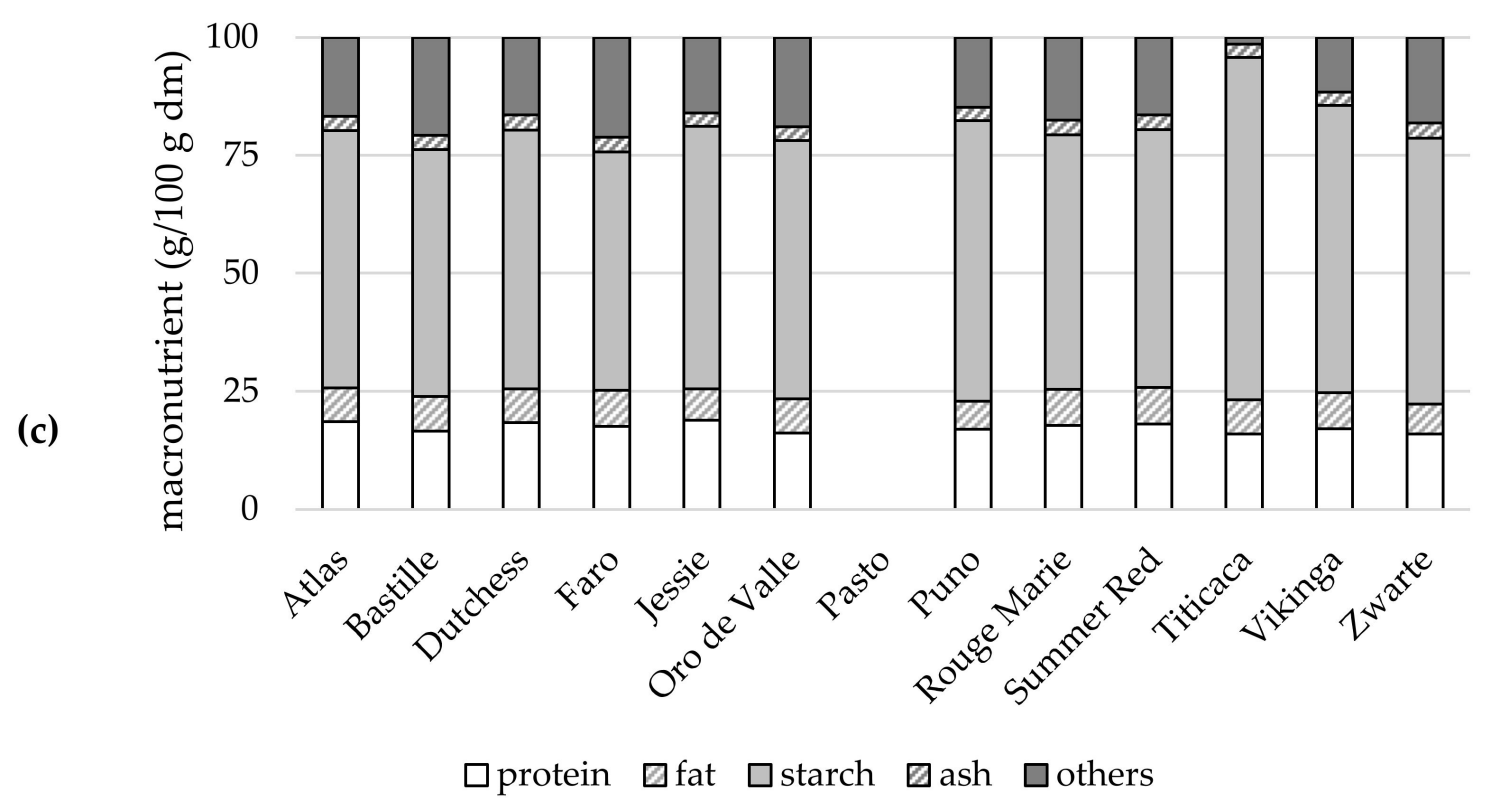
Macronutrient composition (g/100 g dm) of 13 quinoa varieties grown under North-West European field conditions in 2017 (**a**), 2018 (**b**), and 2019 (**c**) (*n* = 3).

The macronutrient composition (Table S2) of the quinoa seeds varied with variety and growing season, with a significant interaction effect between both factors. Macronutrient composition is presented in Figure 2 and discussed in the following paragraphs.

#### 2.5.2. Protein and Amino Acids

The protein content ranged from 12.1 to 18.8 g/100 g dry matter (dm) (Table S2) and was strongly year-dependent, as previously reported by Granado-Rodríguez et al. [19]. The protein contents determined in this study were similar to those reported by Bhargava et al. (12.6–21.0 g/100 g, [17]) and Granado-Rodríguez et al. (13.8–19.1 g/100 g, [19]). Lower values were achieved by Präger et al. (11.9–16.1 g/100 g, [4]) and Miranda et al. (11.3–16.1 g/100 g dm, [23]). The higher protein content of quinoa, compared to other grain staple crops, is an important nutritional fact [12].

All varieties achieved the highest protein concentration in 2019 (16.0–18.8 g/100 g dm). In spite of the 35 units higher nitrogen fertilization in 2017, the protein concentrations did not reach those of 2019. The extra nitrogen fertilization was probably insufficiently available for the plants during growth due to the extremely dry spring in 2017 (Figure 5). A negative trend was observed between yield and protein content. However, this trend was only significant for 2019 (*r* = −0.690, *p* = 0.020). This negative correlation between yield and protein content was also reported by Präger et al. [4] and Granado-Rodríguez et al. [19]. In 2017 and 2018, Oro de Valle and Atlas showed stable protein contents around 12.1 and 16.5 g/100 g dm, respectively. Compared to 2017, the protein content of Pasto and Dutchess increased by 3.1% and 4.3% in 2018, whereas the other varieties showed a reduction in protein content. Averaged over 2017–2019, Atlas and Pasto showed a significantly higher protein content compared to other varieties. In 2018, these varieties had a similar protein content, but in other years, the protein portion was higher in Atlas seeds. In 2017 and 2019, Jessie was also characterized by high protein levels. According to the findings of Rodríguez Gómez et al. [33], Atlas, Pasto, and Jessie were seeds with similar protein content. In 2017 and 2019, Jessie had a protein content comparable to that of Pasto and Atlas, respectively. Both Präger et al. [4] and Granado-Rodríguez et al. [19] concluded that Puno and Titicaca seeds had similar protein levels, even over different growing seasons. However, Aluwi et al. [16] reported a higher protein content for Puno, which corresponded with the results from 2019 within the present study. Nevertheless, the protein content of Titicaca was higher compared to that of Puno in 2017 and 2018. The Farm Original varieties, except for Faro, were characterized by lower average protein content. Aluwi et al. [16] also classified Oro de Valle and Black (possible origin for variety Zwarte) as varieties with a lower protein content than Titicaca or Puno. In 2017 and 2018, the protein content of Oro de Valle and Zwarte ranged between 12.1 and 13.0 g/100 g dm. However, the protein content increased to 16.0–16.2 g/100 g dm in 2019, which reduced the gap with the values of the European-bred varieties (16.0–18.8 g/100 g dm). The other Farm Original variety Faro had medium-high protein content over the different growing seasons (15.2 g/100 g dm). The protein content showed distinct genetic variation. However, yearly differences in protein content can reduce these differences greatly, proving that agro-ecological conditions are an important factor too, as was previously reported by other studies [4,15,19,34]. 

In addition to protein quantity, amino acid composition is an important property for the evaluation of protein quality [34]. Quinoa seeds from the different growing seasons contained large amounts (76–183 mg/g protein) of glutamic acid, aspartic acid, and arginine, along with lesser amounts (42–98 mg/g protein) of leucine, valine, glycine, and lysine, constituting about 60% of the total amino acids (Tables S3 and S4). This is in accordance with the study of Gonzalez et al. [34]. Specifically, the essential amino acids were available in considerable amounts. In most plant proteins, these amino acids are present in insufficient quantities for a balanced diet [34]. Over the years, the total essential amino acid content has ranged from 338 to 443 mg/g protein (Table S4). Across varieties and growing seasons, lower ranges for total essential amino acids were reported during field trials in Germany (204–278 mg/g protein, [4]). Moreover, the content of total essential amino acids differed in comparison with results from Argentina (180–374 mg/g protein, [34]) or Chile (297–339 mg/g protein, [23]). The main constituents of the essential amino acid profile (Table 2 and Table S4) were leucine (62–98 mg/g protein), lysine (42–73 mg/g protein), and valine (46–64 mg/g protein), as confirmed by previous studies [4,23]. The distribution patterns of the essential amino acids were different among growing seasons yet similar among varieties. However, Präger et al. [4] reported remarkable variations in the composition of the essential amino acid profile across varieties. Reguera et al. [15] noted that varieties grown in Chile presented no differences in amino acid content, while cultivation in Spain resulted in different alanine, asparagine, isoleucine, lysine, and valine contents among varieties. Concentrations of aromatic essential amino acids were considerably higher in 2018 and ranged from 43 to 51 mg/g protein for phenylalanine and from 16 to 24 mg/g protein for tryptophan. In 2018, the levels of methionine and lysine were also higher, while the valine concentrations were reduced during this growing season. The varieties generally had the highest leucine and isoleucine content in 2019. The growth conditions in 2017 resulted in the highest levels of threonine and histidine.

Albumin and globulins make up the main fraction of proteins in quinoa, and they are characterized by a balanced composition of the essential amino acids necessary for a healthy human diet, making quinoa one of the few plants with a complete protein source [12]. This and other studies showed that all essential amino acids were available in sufficient amounts according to the daily requirements, in conformance with FAO/WHO requirements for school kids and adults [12,19,35]. In the category of 6-month-old babies to 3-year-old children, lysine is the limiting amino acid for all varieties and years. Leucine mainly did not meet the standard for this age group in 2018. Small shortages for threonine were also observed for a period of one or two years in a few varieties. However, tryptophan reached the standard for each group in all varieties, in contrast to Granado-Rodríguez et al. [19]. 

#### 2.5.3. Fat, Fatty Acids, and Triacylglycerols

Depending on the growing season, the fat content varied between 5.42 and 8.54 g/100 g dm (Table S2). The results of Pulvento et al. (7.7–7.9 g/100 g dm, [22]) were within the range of the present study, while considerably lower fat contents were reported for a study in Spain (3.90–5.10 g/100 g, [33]). Moreover, the fat content in seeds from Argentina (4.7–7.1 g/100 g dm, [36]) or Chile (2.97–5.65 g/100 g dm, [37]) was also within a lower range. The data showed a significant variety × year effect for fat content in quinoa. The seeds harvested in 2017 generally showed lower fat content compared to the other years of cultivation. However, the growth conditions in 2017 resulted in the highest fat content for Titicaca and Summer Red. In 2017 and 2019, Dutchess and Jessie showed a stable fat content of around 7.10 and 6.70 g/100 g dm, respectively. The quinoa varieties, with the exception of Titicaca and Summer Red, obtained the highest fat content in 2018 (6.37–8.50 g/100 g dm). In 2019, the fat content decreased from 5.2% to 15.6% compared to the growing season of 2018. However, the fat content of Bastille and Titicaca remained stable at around 7.35 and 7.16 g/100 g dm, respectively. Contrary to other varieties, the decrease in 2019 resulted in the lowest fat content among growing seasons for Summer Red. Averaged over the three years, Pasto, Puno, and Zwarte showed significantly lower fat content compared to the other varieties. Summer Red and Vikinga had the highest fat content among varieties, followed by Faro and Rouge Marie. 

Quinoa has a healthy balance between proteins and fat, with an interesting nutritional lipid composition of which essential fatty acids, i.e., polyunsaturated fatty acids (PUFAs), are necessary for a healthy human diet [12]. Contrary to the amino acid profile, the composition of fatty acids varied significantly between varieties. The study by Tang et al. [38] confirmed that the fatty acid profile may vary among varieties grown under the same growth conditions. According to the study of Präger et al. [4], the fatty acid composition did not vary among varieties or years. The fatty acid profile of the quinoa varieties (Table 3 and S5) consisted of 60.4% to 70.6% PUFAs, which corresponded with the results of Rodríguez Gómez et al. (65–70%, [33]). The main PUFA in quinoa seeds was linoleic acid (C18:2 n-6), ranging from 53.0% to 59.8%. Lower values were reported by Tang et al. (44.9–56.6%, [38]) and Vidueiros et al. (43.0–57.5%, [36]). Rodríguez Gómez et al. [33] obtained 60.66% to 61.40% linoleic acid for Jessie, Puno, and Titicaca. The content of linoleic acid was stable over the years yet varied among the varieties. The lowest linoleic acid contents were measured for Bastille, Dutchess, Pasto, and Vikinga and the highest for Puno. Furthermore, the quinoa seeds contained 4.7% to 8.2% α-linolenic acid (C18:3 n-3). This corresponded with the studies of Rodríguez Gómez et al. (5.15–7.17%, [33]), Tang et al. (4.8–9.6%, [38]), and Vidueiros et al. (3.2–9.4%, [36]). Compared to 2019, the content of α-linolenic acid was lower in 2018. The red varieties Rouge Marie and Summer Red were characterized by the lowest α-linolenic acid content over the years. The high content of PUFAs makes quinoa lipids a high-quality edible oil, with a composition similar to that of maize and soybean oil [12]. The monounsaturated fatty acid (MUFA) oleic acid (C18:1c) was the second prominent fatty acid in quinoa seeds, ranging from 15.5% to 22.7%. This fatty acid had a large share in the total content of MUFAs (18.1–25.5%), which explains why the total MUFA content was also the highest in 2018. According to Tang et al. [38], the oleic acid content varied between 15.7% and 28.9%, while Rodríguez Gómez et al. [33] reported a lower range of 17.93% to 20.54%. Even higher percentages were obtained by Vidueiros et al. (19.8–33.1%, [36]). Comparing the varieties, Puno had both the lowest oleic acid and total MUFA content. Furthermore, the fat fraction of quinoa contained important amounts of docosadienoic acid (C22:2, 1.3–7.5%), eicosenoic acid (C20:1, 0.9–1.4%), and erucic acid (C22:1, 0.9–1.4%) (Table S5). The balance between Ω6 and Ω3 fatty acids is highly important in health risk reduction, and an optimal ratio should range between 1:1 and 4:1 [39]. Excessive amounts of Ω6 PUFAs and high Ω6/Ω3 ratios promote the pathogenesis of many diseases, including cardiovascular disease, cancer, and inflammatory and autoimmune diseases, whereas increased levels of Ω3 PUFAs (low Ω6/Ω3 ratio) exert suppressive effects [40]. The Ω6/Ω3 ratio of the fat fraction varied between 6.7 and 12.0 (Table S5), which is higher compared to the results of Tang et al. (5.3–10.6, [38]) but within the range reported by Vidueiros et al. (4.9–17.4, [36]). Moreover, these ratios are not within the optimal range to reduce health risk [39]. The growing season of 2018 resulted in the highest Ω6/Ω3 ratios. Among varieties, the highest Ω6/Ω3 ratios were measured for the red varieties Rouge Marie and Summer Red.

Only 10.2% to 15.0% of the total fatty acid content (Table S5) were saturated fatty acids (SFAs). Compared to 2017, the content of SFAs was higher in 2018. The highest SFA contents were found in Titicaca or Vikinga seeds, while Jessie and Faro contained the least SFAs. The most abundant SFAs in quinoa seeds were palmitic acid (C16:0) and stearic acid (C18:0) (Table 3). The levels of palmitic acid ranged between 8.6% and 13.1% and did not vary among growing seasons. According to Vidueiros et al. [36], the level of palmitic acid varied between 8.4% to 22.9%, while Rodríguez Gómez et al. [33] reported 9.02% to 10.37% of palmitic acid. Vikinga and Titicaca contained the highest amount of palmitic acid and Faro the lowest. The levels of stearic acid were considerably lower compared to those of palmitic acid and varied between 0.4% and 1.0%. The highest amounts of stearic acid were found in the lipid fractions of Pasto, Atlas, and Zwarte, while Puno contained the lowest amount of stearic acid. 

The nutritional quality of the fat fraction is defined by its fatty acid composition. However, the triacylglycerol (TAG) composition will determine the physical and functional properties of the fat [41]. Table 4 and Table S6 give the distribution of the different TAGs in the fat fraction of the quinoa varieties. The predominant TAGs were LLO (19.14–32.24 area %), LLL (16.62–26.20 area %), LLP (9.45–13.93 area %), OOL (5.35–10.22 area %), OLP (5.78–9.85 area %), and LLLn (5.04–9.61 area %), where L is linoleic acid, O is oleic acid, P is palmitic acid, and Ln is linolenic acid. According to Jahaniaval et al. [42], the predominant TAGs in the quinoa fat fraction were LLO + OOLn (21.09 area %), LLL (19.22 area %), OOL + PoOO (12.78 area %), LLP + PLnO (11.34 area %), OLP (9.47 area %), and LLLn (6.04 area %). These values were in agreement with the ranges reported in the present study. Fanali et al. [41] identified LLO (18.10 area %), OOL (15.30 area %), OLP (12.93 area %), LLP (8.71 area %), LLL (6.22 area %), and OLLn (5.91 area %) as predominant TAGs. The reported levels of OOL, OLP, and OLLn were higher compared to those described in the present study, while the LLL and LLLn levels were lower [41]. The levels of LLL, LLnP, LLO, LLP, OLP, and PPL showed no differences among the varieties but varied among growing seasons. The levels of LLP, OLP, and PPL were significantly lower in 2017, while the LLL and LLO levels were higher. The highest levels of LLnP were measured in 2019. The content of LLLn, OLLn, and OOL showed more variation among the varieties. Puno was characterized by the highest levels of LLLn but had the lowest share of OOL. Summer Red contained the lowest levels of LLLn and OLLn.

#### 2.5.4. Starch

The quinoa seeds contained 50.5 to 72.5 g starch per 100 g dm (Table S2). Aluwi et al. [16] reported a starch content of 54.4 to 64.3 g/100 g dm. Most varieties obtained the highest starch content in 2018, with the exception of Summer Red, Oro de Valle, and Titicaca. The growth conditions in 2017 resulted in the highest starch content for Summer Red and Oro de Valle, while the amount of starch was the highest in 2019 for Titicaca. The starch content of Atlas seeds remained stable at around 57.8 g/100 g dm in 2017 and 2018 and reduced by 5.7% in 2019. The seeds of 2019 generally had the lowest starch content, although Jessie, Titicaca, Vikinga, and Zwarte seeds contained the lowest amount of starch in 2017. Averaged over the different years, Titicaca seeds were characterized by the highest starch content. The Farm Original variety Faro generally had the lowest starch content among varieties. Other Farm Original varieties had medium-high (Zwarte) to high (Oro de Valle) starch content.

#### 2.5.5. Minerals

The ash content of the quinoa varieties ranged between 2.37 and 3.60 g/100 g dm (Table S2). A higher total mineral content was reported by Pulvento et al. (3.96–4.28 g/100 g dm, [22]), while Rodríguez Gómez et al. [33] obtained an ash content of 2.99 to 3.80 g/100 g. The test weight correlated negatively with the ash content of the seeds from 2017 (*r* = −0.734, *p* = 0.010) and 2018 (*r* = −0.695, *p* = 0.018). The growth conditions of 2017 resulted in the lowest ash content for Puno, Oro de Valle, Pasto, and Zwarte, while other varieties obtained the lowest ash content in 2019. The ash content of Faro seeds remained stable in 2018 and 2019, while Dutchess and Summer Red had a stable ash content in 2017 and 2018. The highest amounts of minerals were found in the seeds of 2018. Only Atlas, Faro, and Jessie contained higher levels of minerals in 2017. In 2018, ash content correlated positively with the seed protein content (*r* = 0.768, *p* = 0.006). Among varieties, Puno invariably had the lowest ash content. Pasto was characterized by the highest amount of minerals, followed by Dutchess, Atlas, and Summer Red. According to Rodríguez Gómez et al. [33], the ash content of Atlas, Jessie, and Pasto seeds was similar among varieties. Within the present study, Jessie invariably had a lower ash content compared to Atlas or Pasto. Varieties Black (possible origin for Zwarte), Oro de Valle, Puno, and Titicaca were included in the study by Aluwi et al. [16]. The authors concluded that Oro de Valle had the lowest ash content among these four varieties [16]. Within the present study, Oro de Valle generally had a higher ash content than Puno or Titicaca, but Zwarte invariably had the highest ash content among these varieties. Titicaca seeds had a lower ash content than Zwarte seeds, although Aluwi et al. [16] reported that these varieties had comparable ash content.

The quinoa seeds (Table 5 and Table S7) showed a high content of potassium (K, 8790–14053 mg/kg dm), phosphorus (P, 3720–6156 mg/kg dm), magnesium (Mg, 1866–2742 mg/kg dm), and calcium (Ca, 399–806 mg/kg dm). Rodríguez Gómez et al. [33] reported similar levels of K (9088–13236 mg/kg dm), P (5162–5829 mg/kg dm), and Mg (1823–2319 mg/kg dm) but higher levels of Ca (868–1035 mg/kg dm) for quinoa seeds cultivated in Spain. Furthermore, the authors concluded that varieties Atlas, Jessie, and Pasto contained similar levels of P, Ca, and Mg. However, Jessie was characterized by higher levels of K [33]. Within the present study, Pasto contained higher Ca and Mg levels compared to Atlas and Jessie, but its P content was lower. The K concentrations were higher in Pasto than in Jessie. Granado-Rodríguez et al. [19] analyzed Puno, Titicaca, and Vikinga seeds cultivated over three growing seasons in Spain. Over the years, these varieties did not differ based on their P, K, Ca, and Mg levels. However, Vikinga had a lower P content in 2017 compared to Titicaca and Puno [19]. The present data confirmed that Puno, Titicaca, and Vikinga seeds had similar P, K, Ca, and Mg concentrations over different years. According to Miranda et al. [37], Chilean quinoa stored considerable higher amounts of K (10638–27103 mg/kg dm) and Ca (1108–3020 mg/kg dm) but lower levels of P (2888–4602 mg/kg dm) and Mg (1648–1768 mg/kg dm). All these data suggest that the mineral content in quinoa is both variety-dependent and the result of environmental conditions, such as soil type and its mineral composition, the used fertilization, and growing season [33]. The levels of P and Ca in this study did not differ over the years but varied between the varieties. The dark-colored varieties (i.e., Rouge Marie, Summer Red, and Zwarte) and Pasto accumulated the highest levels of P; Puno and Titicaca accumulated the lowest. Dutchess, Faro, and Pasto had a higher Ca concentration compared to Summer Red and Titicaca. The P and Mg concentrations were higher in the seeds of 2018, but no differences between varieties were observed. However, Pasto seeds tended to contain higher levels of Mg compared to other varieties cultivated in 2017 or 2018. The iron (Fe) content of the quinoa seeds showed no significant effect of variety or year, while the sodium (Na) content was only affected by the year (Table 5 and S7). Granado-Rodríguez et al. [19] also observed no significant differences between varieties Puno, Titicaca, and Vikinga in the Fe or Na content. In 2017 and 2018, several varieties showed a remarkably higher Fe or Na content compared to other years or varieties, which suggests a significant variety × year interaction. The Fe content generally ranged between 50 and 91 mg/kg dm, but higher levels, up to 723 mg/kg dm, were occasionally measured in 2017 and 2018. Rodríguez Gómez et al. [33] obtained an Fe content of 72 to 94 mg/kg dm and concluded that Atlas, Jessie, and Pasto seeds contained similar levels of Fe. However, the present data showed that Pasto had remarkably higher Fe content than Atlas and Jessie in 2017. A wider range for Fe content was reported by Miranda et al. (57–342 mg/kg dm, [37]). The Na concentration varied from 42 to 165 mg/kg dm, which corresponded with the study by Rodríguez Gómez et al. (74–159 mg/kg dm, [33]). However, the Na content was considerably higher for Dutchess (297 mg/kg dm) and Zwarte (241 mg/kg dm) in 2017 and for Puno (226 mg/kg dm) in 2018. Rodríguez Gómez et al. [33] observed that Atlas seeds contained higher levels of Na than Pasto seeds. The opposite applied for the growing season of 2017 within the present study. The zinc (Zn) concentration ranged between 38 and 109 mg/kg dm (Table 5 and S7), with the highest levels measured for Atlas, Pasto, and Bastille. The results of Miranda et al. (76.7–95.6 mg/kg dm, [37]) were within this range. Furthermore, the quinoa seeds (Table S7) contained low levels of copper (Cu, 2–19 mg/kg dm), boron (B, 9–18 mg/kg dm), and manganese (Mn, 19–46 mg/kg dm). The Cu content did not vary among varieties or years, only Atlas showed a remarkably higher Cu concentration in 2018 (179 mg/kg dm). The varieties contained a similar level of B but the concentrations were slightly higher in 2019. The levels of Mn were higher in seeds from 2018 or in Pasto seeds. 

As described, the effects of variety or year were not always present for the different minerals. Except for Na, Granado-Rodríguez et al. [19] did report significant effects of both variety and year on the mineral content of quinoa. Except for Zn, Reguera et al. [15] also described a significant effect of the growth location. Besides proteins and fats, minerals are an essential quality of quinoa seeds. The mineral content for Ca, Mg, Fe, Cu, and Zn are higher than in common cereals and are, in this study, except for Ca, similar or higher than those reviewed by Bhargava et al. [12]. The minerals Ca, Mg, Fe, and K are present in sufficient amounts and available for a healthy human diet in bioavailable forms [19].

### 2.6. Principal Components Analysis and Hierarchical Cluster Analysis

**Figure 3:**
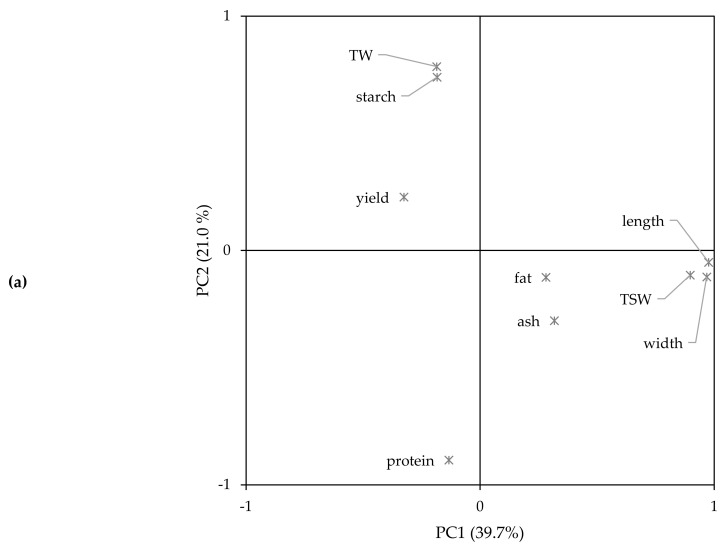

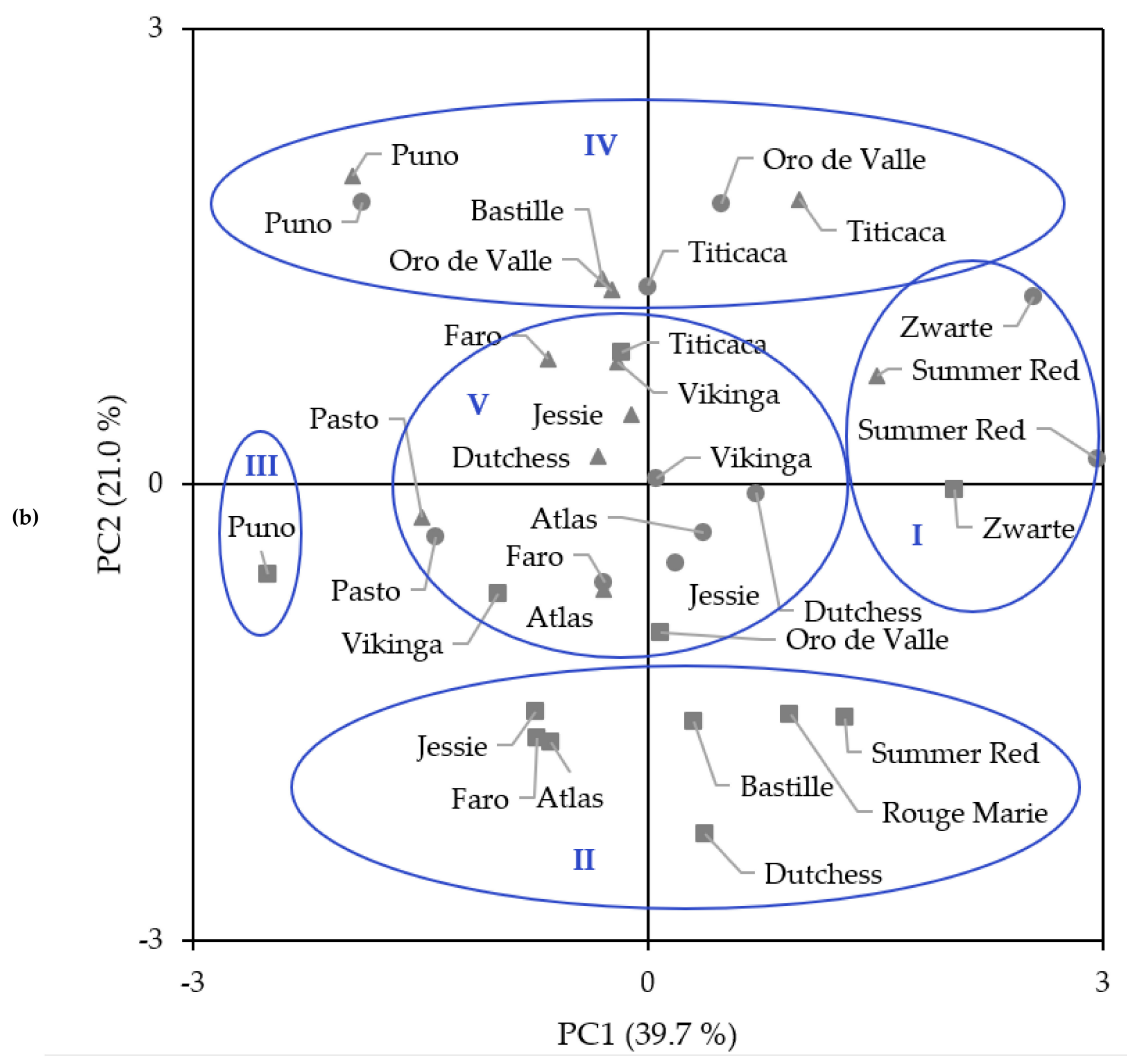
Principal components analysis (PCA—(**a**): loading plot, (**b**): score plot) of yield, seed characteristics, and composition of 13 quinoa varieties grown under North-West European field conditions in 2017 (●), 2018 (▲), and 2019 (■). TSW: thousand seed weight, TW: test weight.

For a more comprehensive understanding of the variation among quinoa seeds, a PCA was performed on yield, seed characteristics (seed size, TSW, test weight), and macronutrient composition (protein, fat, starch, ash). The PCA revealed three principal components (PCs) with eigenvalues greater than one, which explained 74.5% of the total variance. The loading plot of the first two PCs and the score plot with the varieties/years are illustrated in Figure 3. The first and second PCs accounted for 39.7% and 21.0% of the variance, respectively. The first PC was defined by seed length and width and TSW (Table S8). These were all properties related to the physical properties of the seed, of which the TSW correlated positively with seed length (*r* = 0.873, *p* < 0.001) and width (*r* = 0.854, *p* < 0.001). A strong positive association between TSW and seed size was also reported by Bhargava et al. [17]. The second PC was defined by test weight and protein and starch content (Table S8). The protein content correlated negatively with the starch content (*r* = −0.543, *p* = 0.001) and test weight (*r* = −0.607, *p* < 0.001). Protein-rich seeds showed a lower starch content, which suggests that the share of perisperm is smaller in these seeds. The smaller ratio of perisperm to bran and embryo could affect the test weight of the seeds, explaining the negative relation between the protein content and test weight [43,44]. The third PC explained 13.8% of the variance and was representative for the yield, ash, and fat content (Table S8). 

Based on the yield, seed characteristics, and macronutrient composition, the varieties/years were classified into five groups (I–V) in the score plot (Figure 3b). Group I had the highest positive score on PC1 and included Summer Red of 2017 and 2018 and Zwarte of 2017 and 2019. These varieties were, within the given growing season, characterized by the largest seeds with the highest TSW. Group II only included varieties of 2019, namely, Atlas, Bastille, Dutchess, Faro, Jessie, Rouge Marie, and Summer Red. This growing season was characterized by protein-rich seeds with low test weight. However, the seed size and TSW of these varieties were diverse, as seen in the spread in scores on PC1. Puno 2019 had a high negative score on PC1 due to the small size of the seeds and was, therefore, included in Group III. The varieties in Group IV had a high positive score on PC2, indicating high test weight and starch content. This group included Bastille 2018, Oro de Valle 2017 and 2018, Puno 2017 and 2018, and Titicaca 2017 and 2018. The remaining varieties were classified in Group V and were, in fact, all close to the origin of the score plot.

**Figure 4:**
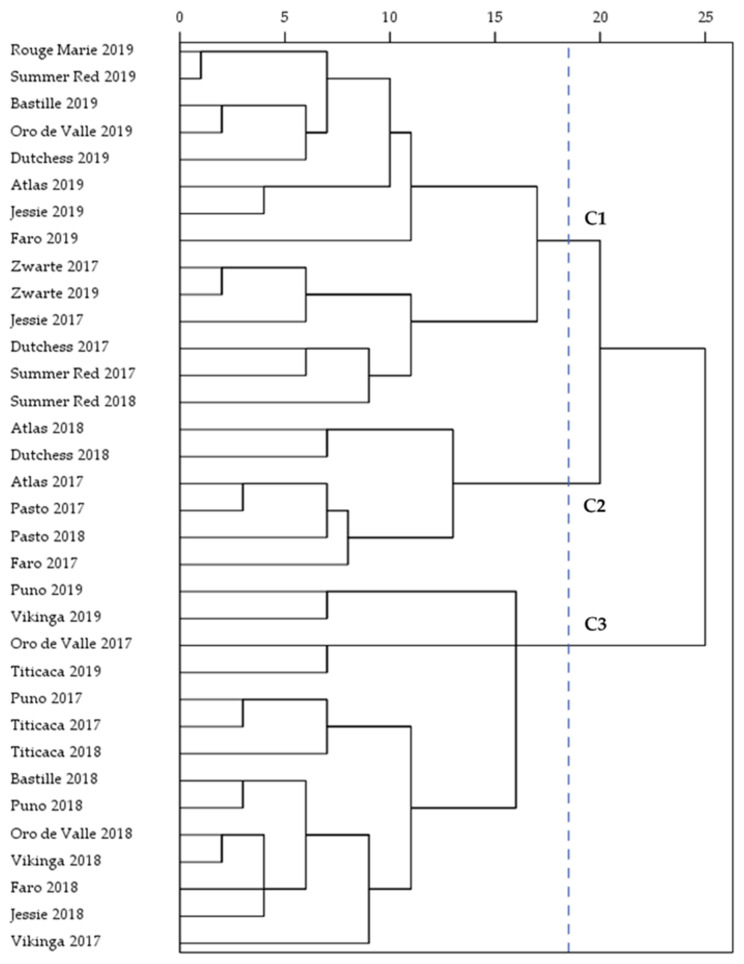
Dendrogram of the three clusters (C1–C3) based on hierarchical clustering.

A total of three clusters was identified based on the yield, seed characteristics, and macronutrient composition of the quinoa seeds by using HCA. The dendrogram of the hierarchical clustering is presented in Figure 4. The clustering revealed subtle differences between the growing seasons as most varieties of 2019 were grouped in the first cluster. However, the factor variety had a large impact on the clustering of Puno, Rouge Marie, Summer Red, Titicaca, Vikinga, and Zwarte. Hence, the significant interaction effects of year and variety were also reflected in the clustering of the varieties. Atlas, Bastille, Faro, and Oro de Valle seeds of 2019 showed more similarities to varieties of the same growing season than to seeds of the same variety from a different growing season and were grouped in the first cluster. Cluster 1 also included all dark-colored seeds (i.e., Rouge Marie, Summer Red, and Zwarte) and the seeds of Dutchess and Jessie grown in 2017 and 2019. Rouge Marie, Summer Red, and Zwarte were low-yielding over the years but provided large seeds with a high TSW. The second cluster was a small cluster (*n* = 6), only including seeds of 2017 and 2018. Atlas and Pasto seeds of the first two growing seasons were included in this cluster. Furthermore, Dutchess seeds of 2018 and Faro seeds of 2017 were grouped in the second cluster. All Puno, Titicaca, and Vikinga seeds were part of the third cluster. Titicaca and Vikinga were high-yielding varieties, while variety Puno produced small seeds with low levels of fat and ash. The Oro de Valle seeds of 2017 and 2018 were also grouped in the third cluster, as well as the Bastille, Faro, and Jessie seeds of 2018. The growing season helped to spread the seeds of Faro and Bastille over the three different groups, indicating a strong impact of environmental conditions.

## 3. Materials and Methods

### 3.1. Plant Varieties

Ten different quinoa (*Chenopodium quinoa* Willd.) varieties were obtained from commercial companies (Gilbel sprl, Saint-Georges-Sur-Meuse, Belgium (previously a sublicensee of Radicle Crops, Wageningen, The Netherlands) and Quinoa Quality ApS, Regstrup, Denmark) and three Farm Original varieties were purchased at De Nieuwe Tuin (De Klinge, Belgium). Besides white varieties, the set included two red varieties (i.e., Rouge Marie and Summer Red) and one black variety (i.e., Zwarte). An overview of the main characteristics of these varieties is listed in Table 6. The varieties of Atlas, Dutchess, and Pasto were bred at Plant Breeding, Wageningen Research (Wageningen, The Netherlands), and Bastille, Jessie, and Rouge Marie were bred by AbbottAgra (Longué-Jumelles, France). These varieties were area-adapted to the West European climate and photoperiod. Summer Red is the predecessor of Rouge Marie, bred by AbottAgra. Puno, Titicaca, and Vikinga were derived from crosses between Chilean and Peruvian material, followed by selection for long day length in Denmark [32]. The Farm Original varieties (i.e., Faro, Oro de Valle, and Zwarte) were adapted to/selected for the Belgian climate by De Nieuwe Tuin.

### 3.2. Field Trials

**Figure 5:**
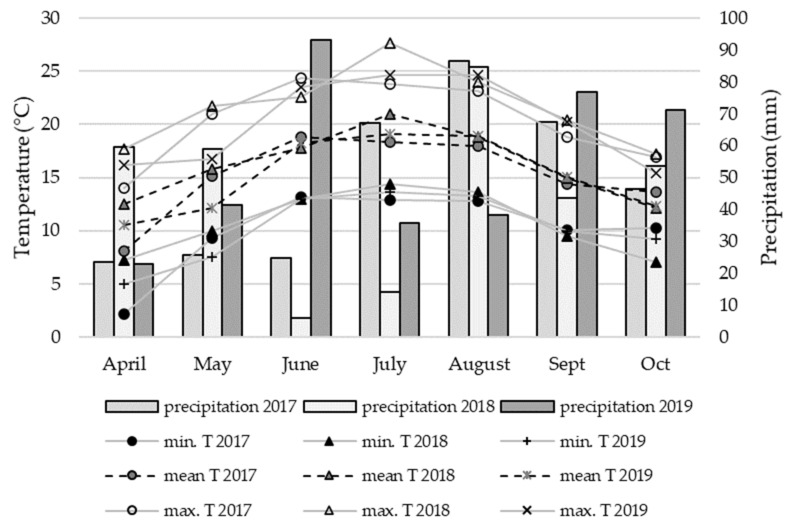
Total monthly precipitation (mm) and minimum, mean, and maximum monthly temperature (T, °C) during the experimental period in 2017, 2018, and 2019.

Agronomy tests were conducted in a sandy loam soil on ILVO trial fields in Melle/Merelbeke, Belgium (50°58′52.39″ N, 3°46′34.605″ E). The varieties were sown with a Jacobi seeder in three (2017) or four repetitions (2018 and 2019) per variety on 12 April (2017), 24 April (2018), and 26 April (2019). The repetitions were arranged in blocks, with full randomization within the block. The field plots were 13 m^2^ in size. The average temperature and precipitation during the growth period were 15.2 °C and 342 mm in 2017, 16.2 °C and 320 mm in 2018, and 15.2 °C and 379 mm in 2019 (Figure 5), respectively. The field plots were not irrigated. Maximum temperatures were generally higher in 2018, except for June and August. The precipitation in June and July was considerably lower in 2018 than in 2017 and 2019. Meteorological data were collected by the Royal Meteorological Institute of Belgium (RMI).

Sowing density was adjusted per seed lot to obtain a (theoretical) sowing density of 400 (2017 and 2018) or 300 (2019) germinating seeds per m^2^. The inter-row distance was 26 cm, and the sowing depth was 1 to 2 cm. Calcium ammonium nitrate was applied before sowing at a rate of 135 units nitrogen in 2017 and 100 units nitrogen in 2018 and 2019. Weeding was done manually when necessary. Harvesting of the separate plots was done with a Wintersteiger harvester (Ried im Innkreis, Austria) according to the senescence stage of each variety (BBCH stage 95). After harvesting, seeds from each plot were collected in separate bags and dried using ventilation at 30 °C, cleaned with a Westrup LA-LS (Slagelse, Denmark), and color-sorted with a SEA Chromex (Cimbria, Thisted, Denmark) in 2017 and 2018 and an Os F color sorter (Petkus Selecta, Wutha-Farnroda, Germany) in 2019. Yield was determined after color sorting. Cleaned seeds were kept in a short storage room at 16 °C. 

### 3.3. Seed Characteristics

Test weight (kg/hL) was determined by a grain analysis computer (GAC 2100, Dickey-john, Auburn, AL, USA). TSW (g) was calculated based on the weight of 300 seeds selected by a Contador seed counter (Pfeuffer GmbH, Kitzingen, Germany). Seed color, expressed as CIELAB coordinates L* (lightness), a* (redness to greenness), and b* (yellowness to blueness), was measured using a Konica Minolta CM-700d spectrophotometer (Konica Minolta, Tokyo, Japan). Seeds were scanned (dpi = 600) against a blue background (HP Scanjet 2400, Hewlett-Packard Company, Palo Alto, CA, USA). The resulting images were analyzed using SmartGRAIN software (version 1.1, National Institute of Agrobiological Sciences, Tsukuba, Japan) to estimate the length (mm), width (mm), and LWR of the seeds. The saponin content of the seeds was estimated with Koziol’s standard afrosimetric foam test [26] and calculated according to the following formula (1):

(1) $$\mathrm{saponin}\;\left(\mathrm{mg}/g\right)=\frac{0.646\times H-0.104}{W}$$

where ‘H’ is the foam height in cm, and ‘W’ is the sample weight in g [26]. All seed characteristics were analyzed in triplicate.

### 3.4. Chemical Composition

For chemical analyses, seeds were milled to wholemeal flour (WMF) by a Hammertec mill (mesh size: 0.8 mm) (Foss, Hilleroed, Denmark).

#### 3.4.1. Macronutrients

Moisture (g/100 g) and ash (g/100 g dm) content were determined according to ICC methods no. 110 and 104/1, respectively. Total starch content (g/100 g dm) was analyzed as described by Englyst et al. [46]. The nitrogen content was determined using aVarioMax C/N (Elementar Analysesystemen, Langenselbold, Germany) and converted to protein content (g/100 g dm) using a conversion factor of 6.25 [47]. Fat content (g/100 g dm) was determined by Soxhlet extraction with prior acid hydrolysis (ISO 6491). All macronutrient analyses were performed in triplicate.

#### 3.4.2. Amino Acids

To determine the total standard amino acid composition (excluding tryptophan), acid hydrolysis was performed on the lyophilized samples using a hydrogen chloride and dithioglycolic acid solution for 4 h at 150 °C. Sample purification was done on an OASIS HLB cartridge (200 mg, 6 cc), followed by separation and detection by an LC–MS2 instrument (Nexera 8040, Shimadzu, Kyoto, Japan). Separation was done in normal phase mode on an Intrada amino acid column (3.0 μm, 100 × 3.0 mm) with acetonitrile (0.3% acetic acid) as solvent A and 20% acetonitrile-80% 100 mM ammonium formate as solvent B for the mobile phase gradient. The solvent gradient started at 80% solvent A and 20% solvent B. After 4 min, solvent B was gradually increased to 100% until the 14th min, at which it was held for 2 min before being returned to the initial gradient for 9 min. Amino acids were ionized by electrospray ionization (4.5 kV) followed by multiple reaction monitoring (MRM) on a triple quadrupole. All amino acids were measured in positive mode, except for aspartic acid, which was measured in negative mode. Methylvaline, homoarginine, and methyl-D3-methionine were used as internal standards.

To determine the tryptophan, alkaline hydrolysis was performed on lyophilized samples using a lithium hydroxide solution for 4 h at 150 °C. Sample purification was done on an OASIS HLB cartridge (200 mg, 6 cc), followed by separation and detection by an LC-MS2 instrument (Nexera 8040, Shimadzu Corporation, Kyoto, Japan). Separation was done in normal phase mode on an Intrada amino acid column (3.0 μm, 100 × 3.0 mm) with acetonitrile (0.3% acetic acid) as solvent A and 20% acetonitrile-80% 100 mM ammonium formate as solvent B for the mobile phase gradient. The solvent gradient started at 80% solvent A and 20% solvent B. After 4 min, solvent B was gradually increased to 100% until the 14th min, at which it was held for 2 min before being returned to the initial gradient for 9 min. Amino acids were ionized by electrospray ionization (4.5 kV), followed by MRM on a triple quadrupole. Tryptophan was measured in positive mode with methyl-tryptophan as the internal standard.

#### 3.4.3. Fatty Acids

The fatty acid profile of the quinoa samples was determined as described by Foubert et al. [48]. Quinoa WMF was mixed with diethyl ether to extract the fat. The mixture was subsequently filtered over sodium sulfate, and diethyl ether was evaporated to retain the extracted fat. Four droplets of extracted quinoa fat were dissolved in 9 mL hexane and 1 mL 2 N potassium hydroxide/methanol reagent to produce fatty acid methyl esters (FAMEs). The blend was shaken for 30 s and allowed to settle. The FAMEs in the hexane layer were separated by a Varian 3380 gas chromatograph (Varian Inc., Palo Alto, CA, USA) equipped with a WCOT CP-sil 88 column and a flame ionization detector (FID). The conditions for the gas-chromatographic analysis were: temperature of injector: 250 °C, temperature of detector: 250 °C, flow rate of mobile phase (helium): 1 mL min^−1^, flow rate of hydrogen: 40 mL min^−1^, flow rate of air: 120 mL min^−1^, injection volume: 1 mL, and column oven temperature: 120 °C for 2 min, followed by heating at 5 °C min^−1^ to 200 °C and holding at that temperature for 20 min.

#### 3.4.4. Triacylglycerols

Analysis of the TAG species was performed as described by Rombaut et al. [49]. Quinoa WMF was mixed with diethyl ether to extract the fat. The mixture was subsequently filtered over sodium sulfate, and diethyl ether was evaporated to retain the extracted fat. The extracted fat was dissolved in a concentration of 5 mg/mL in dichloromethane/acetonitrile (30:70 *v*/*v*). Separation of the TAG species was performed on a Thermo Finnigan Surveyor RP-HPLC system (Thermo Electron Corporation, Brussels, Belgium) using a 150 × 3.0 mm Alltima HP C18 HL column with a 3 μm particle diameter (Grace Alltech, Lokeren, Belgium) and connected to an Alltech ELSD 2000 evaporative laser light scattering detector (Grace Alltech, Lokeren, Belgium). The injection volume was 25 µL.

#### 3.4.5. Minerals

The mineral composition was determined via inductive coupled plasma-optical emission spectrometry (ICP-OES, IRIS Intrepid II XSP, Thermo Scientific, Waltham, MA, USA). Prior to the analysis, 1.0 g of WMF was ashed at 500 °C for 4 h in a muffle furnace. The ash was dissolved in hydrochloric acid during a 2 h reflux. The remaining residue was filtered (Whatman filter no. 5) before analysis.

### 3.5. Statistical Analysis

To determine the differences between varieties, years, and their interactions, the *lme4* package in R (version 4.0.2, R Core Team, Vienna, Austria) was used to fit a (mixed) linear model [50]. The fixed effects were variety and year, while block was considered a random effect. The following base model (2) was considered:

Y = Variety × Year + Random(2)

where ‘Y’ is the response variable, ‘Variety’ is the fixed effect of the variety, ‘Year’ is the fixed effect of the growing season, and ‘Random’ is the random effect of the block or measurement replicate. 

Four different versions (3–6) were derived from the base model, as follows:

Y ~ Random(3)

Y ~ Variety + Random (4)

Y ~ Year + Random(5)

Y ~ Variety + Year + Random(6)

The five versions of the model (2–6) were tested, and the output was evaluated using Akaike information criteria (AIC) [51]. The best fit for each trait was then chosen based on the lowest AIC value (summarized in Table S9). Then, an analysis of variance (ANOVA) was performed, and pairwise comparisons between varieties and growing seasons were performed with the Tukey test. For the analyses of the seed characteristics and composition, one block was analyzed per variety. Therefore, the random effect of the measurement replicates was included in the base model instead of the block effect. In the case where ‘Random’ did not significantly attribute to the model, this effect was excluded from the model.

The analyses of amino acid, fatty acid, TAG, and mineral composition were performed without replicates. Therefore, the data did not allow the study of the effect of variety, year, or variety × year interaction. The data of all varieties common to all years were grouped to be able to make a comparison between the years. In a similar way, the data were grouped over the years to be able to estimate the variety effect.

A PCA and an HCA were performed with SPSS Statistics 27 (SPSS Inc., Chicago, IL, USA). For a more comprehensive understanding of the variation among quinoa varieties, a PCA was performed on the analyses of yield, seed characteristics, and macronutrient composition. Prior to analysis, the suitability of a PCA was assessed by testing the linearity between the variables and sample adequacy. Inspection of the correlation matrix showed that all variables had at least one correlation coefficient greater than 0.3. The overall Kaiser-Meyer-Olkin (KMO) measure was 0.641, with individual KMO measures all greater than 0.5. Bartlett’s test of sphericity was statistically significant (*p* < 0.001), indicating that the data was likely factorizable. PCA revealed three components with eigenvalues greater than one, and visual inspection of the scree plot indicated that all three components should be retained. A Varimax orthogonal rotation with Kaiser normalization was employed to aid interpretability. The data on yield, seed characteristics, and macronutrient composition were used to perform an HCA to group the quinoa varieties with similar properties. The HCA was performed by using the unweighted paired-group method with an arithmetic mean. Distances among clusters were computed using Pearson correlation coefficients.

## 4. Conclusions

Thirteen varieties were evaluated for their agronomic performance under North-West European field conditions during three consecutive growing seasons (2017–2019). Additionally, the seeds were quantitatively characterized based on characteristics and composition. The study showed that all varieties, except for the very late ones, performed well under the environmental conditions in North-West Europe, thereby combining competitive yields with high seed quality over the years. Clustering of the varieties/years revealed subtle differences between growing seasons, but the factor of variety had a large impact on the clustering of Puno, Rouge Marie, Summer Red, Titicaca, Vikinga, and Zwarte. Hence, the significant interaction effects of variety and year were reflected in the clustering of the varieties.

Under North-West European environmental conditions, the studied varieties obtained nutritional values within the range of composition traits previously reported for European quinoa. The seeds contained high protein levels, and the varieties had a similar amino acid profile, containing all essential amino acids. The main fatty acids were linoleic, α-linolenic, and oleic acid, indicating a high degree of unsaturation. As a good source of minerals, the seeds were characterized by high K, P, Ca, and Mg levels and important amounts of Fe and Na. Varieties such as Bastille, Dutchess, Titicaca, and Vikinga were high-yielding and better adapted to North-West European environmental conditions. Atlas and Pasto did not reach full maturity under these environmental conditions but performed best regarding their protein and mineral content. The Farm Original varieties (i.e., Faro, Oro de Valle, and Zwarte) reached yields similar to those of most European-bred varieties. However, Oro de Valle and Zwarte were characterized by the lowest protein levels. Faro showed a more interesting nutritional profile as it was characterized by medium-high protein and high-fat content but was the bitterest variety.

Altogether, this study supports the huge potential of quinoa as a European crop. Thereby, the selection of the varieties should be well-informed and aligned with the production aim. However, these varieties are insufficiently studied to define their potential end-uses and facilitate their introduction in the European food industry. To gain more insight into the potential end-uses of North-West European quinoa, the seeds were milled to wholemeal flour and the physicochemical properties of these flours were determined. The physicochemical properties are described in Part II of this issue. Furthermore, the variability in yield, physical, and nutritional properties showed possibilities to stabilize or improve the yield and quality of European quinoa varieties using suitable agronomic practices. However, more research is needed to optimize these agronomic practices.

## Figures and Tables

**Table 2:** Amino acid composition (mg/g protein) of 13 quinoa varieties grown under North-West European field conditions in 2017, 2018, and 2019.

Variety ^1^/Year ^2^	*n*	Amino Acid (mg/g Protein) ^3^								
		*Phe*	*Leu*	*Met*	*Ile*	*Val*	*Thr*	*His*	*Lys*	*Trp*
Atlas	3	45 ± 3 ^a^	76 ± 9 ^a^	20 ± 3 ^a^	45 ± 8 ^a^	58 ± 15 ^a^	36 ± 3 ^a^	30 ± 3 ^a^	54 ± 7 ^a^	17 ± 2 ^a^
Bastille	2	42 ± 1	71 ± 12	22 ± 2	42 ± 5	53 ± 9	36 ± 1	25 ± 7	51 ± 2	19 ± 5
Dutchess	3	42 ± 4 ^a^	74 ± 6 ^a^	20 ± 3 ^a^	42 ± 5 ^a^	56 ± 13 ^a^	38 ± 6 ^a^	30 ± 3 ^a^	52 ± 4 ^a^	17 ± 2 ^a^
Faro	3	42 ± 6 ^a^	75 ± 4 ^a^	23 ± 3 ^a^	44 ± 2 ^a^	60 ± 7 ^a^	39 ± 4 ^a^	29 ± 5 ^a^	55 ± 4 ^a^	17 ± 2 ^a^
Jessie	3	43 ± 6 ^a^	76 ± 5 ^a^	24 ± 2 ^a^	44 ± 2 ^a^	61 ± 7 ^a^	41 ± 3 ^a^	30 ± 6 ^a^	56 ± 1 ^a^	17 ± 2 ^a^
Oro de Valle	3	43 ± 6 ^a^	74 ± 5 ^a^	24 ± 2 ^a^	43 ± 3 ^a^	60 ± 6 ^a^	41 ± 3 ^a^	30 ± 6 ^a^	57 ± 2 ^a^	15 ± 4 ^a^
Pasto ^4^	2	42 ± 4	70 ± 12	24 ± 1	41 ± 3	58 ± 17	43 ± 11	29 ± 10	59 ± 7	16
Puno	3	44 ± 6 ^a^	74 ± 5 ^a^	26 ± 5 ^a^	42 ± 4 ^a^	59 ± 7 ^a^	41 ± 3 ^a^	30 ± 6 ^a^	58 ± 3 ^a^	15 ± 4 ^a^
Rouge Marie	1	41	82	21	48	64	36	30	46	16
Summer Red	3	44 ± 6 ^a^	74 ± 5 ^a^	25 ± 6 ^a^	42 ± 3 ^a^	59 ± 7 ^a^	40 ± 4 ^a^	30 ± 6 ^a^	57 ± 4 ^a^	15 ± 4 ^a^
Titicaca	3	42 ± 8 ^a^	71 ± 7 ^a^	24 ± 7 ^a^	40 ± 5 ^a^	56 ± 6 ^a^	38 ± 2 ^a^	29 ± 5 ^a^	54 ± 5 ^a^	15 ± 3 ^a^
Vikinga	3	42 ± 7 ^a^	73 ± 7 ^a^	24 ± 6 ^a^	42 ± 6 ^a^	59 ± 4 ^a^	40 ± 4 ^a^	28 ± 5 ^a^	56 ± 7 ^a^	16 ± 4 ^a^
Zwarte	2	36 ± 1	73 ± 1	23 ± 1	39 ± 0	64 ± 2	44 ± 1	32 ± 2	57 ± 3	15 ± 2
										
2017	9	38 ± 3 ^A^	74 ± 6 ^A,B^	23 ± 1 ^A^	41 ± 5 ^A^	63 ± 5 ^B^	43 ± 3 ^B^	34 ± 2 ^C^	57 ± 4 ^A,B^	15 ± 2 ^A^
2018	9	48 ± 2 ^C^	69 ± 5 ^A^	28 ± 5 ^B^	41 ± 3 ^A^	51 ± 6 ^A^	38 ± 3 ^A^	23 ± 2 ^A^	58 ± 4 ^B^	21 ± 2 ^B^
2019	9	43 ± 3 ^B^	81 ± 7 ^B^	22 ± 3 ^A^	47 ± 3 ^B^	65 ± 4 ^B^	36 ± 2 ^A^	32 ± 2 ^B^	53 ± 3 ^A^	15 ± 1 ^A^

^1^ Amino acid content per variety averaged over the different years. ^2^ Amino acid content per year averaged over the nine varieties grown in all three years. ^3^ Lowercase letters compare the varieties grown in all three years; average values followed by the same letter are not significantly different (*p* > 0.05). Capital letters compare the three years; average values followed by the same letter are not significantly different (*p* > 0.05). ^4^ No analysis of tryptophan for Pasto in 2017.

**Table 3:** Fatty acid composition (% fatty acid ethyl esters) of 13 quinoa varieties grown under North-West European field conditions in 2017, 2018, and 2019.

Variety ^1^/Year ^2^	*n*	Fatty Acid (% FAME) ^3,4^				
		*C16:0*	*C18:0*	*C18:1c*	*C18:2 n-6*	*C18:3 n-3*
Atlas	3	9.3 ± 0.5 ^a,b^	0.7 ± 0.1 ^e^	20.9 ± 0.3 ^c^	55.3 ± 0.4 ^a,b^	5.4 ± 0.3 ^a,b^
Bastille	2	9.4 ± 0.1	0.6 ± 0.0	19.9 ± 0.6	54.6 ± 0.2	7.9 ± 0.3
Dutchess	3	9.4 ± 0.6 ^a,b^	0.6 ± 0.1 ^d,e^	20.6 ± 0.7 ^c^	54.6 ± 1.0 ^a^	5.6 ± 0.4 ^a,b^
Faro	3	8.9 ± 0.1 ^a^	0.6 ± 0.1 ^c,d,e^	19.9 ± 0.8 ^b,c^	57.4 ± 0.6 ^c^	6.1 ± 0.7 ^b,c^
Jessie	3	9.1 ± 0.1 ^a,b^	0.5 ± 0.0 ^a,b^	18.7 ± 1.3 ^b^	57.3 ± 0.5 ^c^	6.3 ± 0.7 ^b,c^
Oro de Valle	3	9.1 ± 0.3 ^a,b^	0.5 ± 0.0 ^a,b,c^	20.6 ± 0.3 ^c^	56.3 ± 0.9 ^b,c^	6.7 ± 0.2 ^c,d^
Pasto	2	9.6 ± 0.2	0.8 ± 0.2	19.4 ± 0.2	54.8 ± 0.7	6.8 ± 0.3
Puno	3	9.2 ± 0.3 ^a,b^	0.5 ± 0.0 ^a^	16.8 ± 1.2 ^a^	59.0 ± 0.6 ^d^	7.2 ± 0.4 ^d^
Rouge Marie	1	10.0	0.6	19.7	56.3	4.8
Summer Red	3	9.5 ± 0.7 ^a,b^	0.6 ± 0.1 ^a,b,c,d,e^	20.2 ± 1.1 ^b,c^	55.8 ± 0.7 ^a,b^	4.8 ± 0.2 ^a^
Titicaca	3	10.3 ± 1.4 ^b^	0.6 ± 0.1 ^b,c,d,e^	19.7 ± 0.8 ^b,c^	56.7 ± 0.9 ^b,c^	5.5 ± 0.6 ^a,b^
Vikinga	3	10.4 ± 1.2 ^b^	0.6 ± 0.1 ^a,b,c,d^	21.2 ± 1.0 ^c^	54.7 ± 0.9 ^a^	5.8 ± 0.7 ^b,c^
Zwarte	2	9.5 ± 0.2	0.7 ± 0.1	18.9 ± 0.5	56.6 ± 0.6	7.1 ± 0.0
						
2017	9	9.2 ± 0.4 ^A^	0.6 ± 0.1 ^B^	19.6 ± 1.3 ^A^	55.9 ± 1.6 ^A^	6.0 ± 0.7 ^A,B^
2018	9	9.8 ± 1.2 ^A^	0.6 ± 0.1 ^A,B^	20.8 ± 1.2 ^B^	56.4 ± 1.5 ^A^	5.5 ± 0.8 ^A^
2019	9	9.5 ± 0.4 ^A^	0.5 ± 0.1 ^A^	19.2 ± 1.6 ^A^	56.8 ± 1.4 ^A^	6.3 ± 0.8 ^B^

^1^ Fatty acid content per variety averaged over the different years. ^2^ Fatty acid content per year averaged over the nine varieties grown in all three years. ^3^ Lowercase letters compare the varieties grown in all three years; average values followed by the same letter are not significantly different (*p* > 0.05). Capital letters compare the three years; average values followed by the same letter are not significantly different (*p* > 0.05). ^4^ FAME: fatty acid methyl esters.

**Table 4:** Triacylglycerol composition (area %) of 13 quinoa varieties grown under North-West European field conditions in 2017, 2018, and 2019.

Variety ^1^/Year ^2^	*n*	Triacylglycerol (Area %) ^3,4^					
		*LLLn*	*LLL*	*LLO*	*LLP*	*OOL*	*OLP*
Atlas	3	6.08 ± 0.66 ^a,b,c^	20.29 ± 3.46 ^a^	24.00 ± 3.05 ^a^	11.30 ± 1.04 ^a^	8.71 ± 0.71 ^b^	7.74 ± 1.55 ^a^
Bastille	2	7.49 ± 0.54	17.96 ± 1.55	22.04 ± 1.46	11.98 ± 0.26	8.13 ± 0.33	8.34 ± 0.60
Dutchess	3	5.67 ± 0.88 ^a,b^	19.70 ± 3.76 ^a^	25.01 ± 5.65 ^a^	11.53 ± 1.61 ^a^	8.73 ± 0.60 ^b^	8.16 ± 1.89 ^a^
Faro	3	7.11 ± 1.05 ^b,c,d^	20.04 ± 2.21 ^a^	22.23 ± 1.40 ^a^	11.57 ± 0.47 ^a^	8.50 ± 0.59 ^b^	7.78 ± 0.43 ^a^
Jessie	3	7.24 ± 1.08 ^c,d^	20.23 ± 1.62 ^a^	21.88 ± 0.59 ^a^	12.41 ± 0.20 ^a^	7.68 ± 1.10 ^a,b^	8.15 ± 0.81 ^a^
Oro de Valle	3	8.26 ± 1.06 ^d,e^	22.05 ± 1.38 ^a^	22.41 ± 3.12 ^a^	12.02 ± 1.55 ^a^	7.51 ± 1.50 ^a,b^	7.06 ± 0.57 ^a^
Pasto	2	7.47 ± 0.60	19.79 ± 3.28	23.29 ± 3.15	11.47 ± 1.39	7.19 ± 0.52	7.20 ± 1.51
Puno	3	8.92 ± 0.67 ^e^	23.28 ± 0.94 ^a^	21.19 ± 1.33 ^a^	13.18 ± 0.61 ^a^	6.12 ± 0.67 ^a^	6.93 ± 0.53 ^a^
Rouge Marie	1	5.20	18.77	22.37	13.29	8.61	9.35
Summer Red	3	5.30 ± 0.26 ^a^	20.98 ± 4.08 ^a^	24.83 ± 3.31 ^a^	12.36 ± 1.15 ^a^	8.23 ± 1.16 ^b^	8.27 ± 1.94 ^a^
Titicaca	3	6.39 ± 0.58 ^a,b,c^	20.62 ± 0.92 ^a^	22.76 ± 0.62 ^a^	13.16 ± 0.29 ^a^	8.38 ± 0.45 ^b^	8.59 ± 0.47 ^a^
Vikinga	3	6.26 ± 0.90 ^a,b,c^	19.04 ± 1.47 ^a^	23.33 ± 1.68 ^a^	12.08 ± 1.08 ^a^	8.81 ± 1.26 ^b^	8.41 ± 1.48 ^a^
Zwarte	2	8.29 ± 0.28	20.36 ± 1.05	21.93 ± 1.67	12.02 ± 1.13	7.39 ± 0.07	7.63 ± 0.63
							
2017	9	6.98 ± 1.21 ^A,B^	22.36 ± 2.92 ^B^	25.38 ± 3.76 ^B^	11.24 ± 1.28 ^A^	7.65 ± 0.72 ^A^	6.85 ± 1.04 ^A^
2018	9	6.01 ± 1.08 ^A^	19.40 ± 1.73 ^A^	22.52 ± 0.52 ^A^	12.42 ± 0.44 ^B^	8.92 ± 0.91 ^B^	8.87 ± 0.82 ^C^
2019	9	7.41 ± 1.47 ^B^	20.32 ± 2.17 ^A^	21.32 ± 1.28 ^A^	12.87 ± 0.84 ^B^	7.65 ± 1.43 ^A^	7.97 ± 1.01 ^B^

^1^ Triacylglycerol content per variety averaged over the different years. ^2^ Triacylglycerol content per year averaged over the nine varieties grown in all three years. ^3^ Lowercase letters compare the varieties grown in all three years; average values followed by the same letter are not significantly different (*p* > 0.05). Capital letters compare the three years; average values followed by the same letter are not significantly different (*p* > 0.05). ^4^ L: linoleic acid, O: oleic acid, P: palmitic acid, Ln: linolenic acid.

**Table 5:** Mineral composition (mg/kg dm) of 13 quinoa varieties grown under North-West European field conditions in 2017, 2018, and 2019.

Variety ^1^/Year ^2^	*n*	Mineral (mg/kg dm) ^3^						
		*Ca*	*Fe*	*K*	*Mg*	*Na*	*P*	*Zn*
Atlas	3	613 ± 54 ^a,b^	77 ± 3 ^a^	11055 ± 1232 ^a,b^	2299 ± 186 ^a^	97 ± 28 ^a^	5716 ± 370 ^a^	60 ± 4 ^b^
Bastille	2	636 ± 56	392 ± 468	11397 ± 460	2166 ± 138	74 ± 30	4492 ± 410	76 ± 47
Dutchess	3	686 ± 84 ^b^	110 ± 67 ^a^	12055 ± 1233 ^a,b^	2308 ± 123 ^a^	160 ± 129 ^a^	5344 ± 584 ^a^	45 ± 3 ^a^
Faro	3	686 ± 50 ^b^	203 ± 236 ^a^	10634 ± 846 ^a,b^	2486 ± 191 ^a^	73 ± 26 ^a^	5324 ± 560 ^a^	48 ± 5 ^a^
Jessie	3	507 ± 25 ^a,b^	73 ± 8 ^a^	10222 ± 415 ^a,b^	2314 ± 149 ^a^	73 ± 21 ^a^	5533 ± 653 ^a^	47 ± 3 ^a^
Oro de Valle	3	633 ± 59 ^a,b^	85 ± 36 ^a^	11029 ± 537 ^a,b^	2090 ± 241 ^a^	74 ± 18 ^a^	4597 ± 705 ^a^	48 ± 4 ^a^
Pasto	2	777 ± 41	161 ± 99	12539 ± 396	2706 ± 50	122 ± 61	5281 ± 130	67 ± 0
Puno	3	576 ± 70 ^a,b^	66 ± 5 ^a^	9844 ± 1303 ^a^	2164 ± 177 ^a^	124 ± 90 ^a^	4322 ± 633 ^a^	44 ± 1 ^a^
Rouge Marie	1	515	64	12025	2000	57	4766	44
Summer Red	3	474 ± 66 ^a^	58 ± 4 ^a^	12550 ± 409 ^b^	2157 ± 100 ^a^	88 ± 39 ^a^	5103 ± 378 ^a^	44 ± 3 ^a^
Titicaca	3	450 ± 45 ^a^	176 ± 142 ^a^	9869 ± 954 ^a^	2152 ± 152 ^a^	80 ± 24 ^a^	4671 ± 542 ^a^	45 ± 6 ^a^
Vikinga	3	604 ± 97 ^a,b^	73 ± 12 ^a^	10168 ± 399 ^a,b^	2203 ± 106 ^a^	71 ± 17 ^a^	4630 ± 370 ^a^	47 ± 4 ^a^
Zwarte	2	658 ± 107	55 ± 7	13717 ± 476	2049 ± 35	143 ± 138	4515 ± 1	41 ± 2
								
2017	9	564 ± 81 ^A^	101 ± 90 ^A^	11076 ± 1596 ^A^	2197 ± 187 ^A^	137 ± 75 ^B^	4959 ± 608 ^A,B^	48 ± 5 ^A^
2018	9	545 ± 98 ^A^	138 ± 133 ^A^	10259 ± 922 ^A^	2396 ± 139 ^B^	91 ± 22 ^A,B^	5556 ± 444 ^B^	46 ± 5 ^A^
2019	9	634 ± 105 ^A^	69 ± 7 ^A^	11140 ± 764 ^A^	2132 ± 92 ^A^	53 ± 7 ^A^	4564 ± 503 ^A^	49 ± 7 ^A^

^1^ Mineral content per variety averaged over the different years. ^2^ Mineral content per year averaged over the nine varieties grown in all three years. ^3^ Lowercase letters compare the varieties grown in all three years; average values followed by the same letter are not significantly different (*p* > 0.05). Capital letters compare the three years; average values followed by the same letter are not significantly different (*p* > 0.05).

**Table 6:** Overview of quinoa varieties grown in North-West Europe.

Variety	Seed Source ^1^	Origin	Year Data
Atlas	Gilbel	Wageningen University & Research, Wageningen, NL	3
Dutchess	Gilbel	Wageningen University & Research, Wageningen, NL	3
Pasto	Gilbel	Wageningen University & Research, Wageningen, NL	2
Bastille	Gilbel	AbbottAgra, Longué-Jumelles, FR	2
Jessie	Gilbel	AbbottAgra, Longué-Jumelles, FR	3
Rouge Marie	Gilbel	AbbottAgra, Longué-Jumelles, FR	1
Summer Red	Gilbel	AbbottAgra, Longué-Jumelles, FR	3
Puno	Quinoa Quality	University of Copenhagen, Copenhagen, DK [16]	3
Titicaca	Quinoa Quality	University of Copenhagen, Copenhagen, DK [16]	3
Vikinga	Quinoa Quality	University of Copenhagen, Copenhagen, DK [45]	3
Faro	De Nieuwe Tuin	Redwood Seeds, Manton, CA, USA	3
Oro de Valle	De Nieuwe Tuin	Wild Garden Seeds, Philomath, OR, USA [16]	3
Zwarte ^2^	De Nieuwe Tuin	White Mountain Farm, Mosca, CO, USA [16]	2

^1^ Gilbel sprl, Rue du Château d’Eau 54, 4470 Saint-Georges-Sur-Meuse, Belgium; Quinoa Quality ApS, Teglværksvej 10, 4420 Regstrup, Denmark; De Nieuwe Tuin, Trompwegel 27, 9170 De Klinge, Belgium; ^2^ The origin of variety Zwarte is unclear, but since *zwart* is the Dutch translation for black, it is likely that Zwarte originated from variety Black (White Mountain Farm, CO, USA).

## Data Availability

The data presented in this study are available in the article and Supplementary Materials.

## Supplementary Materials

The following are available online at https://www.mdpi.com/article/10.3390/plants10122689/s1, Figure S1. Correlation between yield and growing days (*r* = −0.705, *p* < 0.001) of 13 quinoa varieties grown under North-West European field conditions in 2017, 2018, and 2019, Table S1. Length-width ratio (LWR) and color parameters (L*a*b*) of 13 quinoa varieties grown under North-West European field conditions in 2017, 2018, and 2019 (*n* = 3), Table S2. Macronutrient composition (g/100 g dm) of 13 quinoa varieties grown under North-West European field conditions in 2017, 2018, and 2019 (*n* = 3), Table S3. Non- and semi-essential amino acid composition (mg/g protein) of 13 quinoa varieties grown under North-West European field conditions in 2017, 2018, and 2019 (*n* = 1), Table S4. Essential amino acid composition (mg/g protein) of 13 quinoa varieties grown under North-West European field conditions in 2017, 2018, and 2019 (*n* = 1), Table S5. Fatty acid composition (% fatty acid methyl ester) of 13 quinoa varieties grown under North-West European field conditions in 2017, 2018, and 2019 (*n* = 1), Table S6. Triacylglycerol composition (area %) of 13 quinoa varieties grown under North-West European field conditions in 2017, 2018, and 2019 (*n* = 1), Table S7. Mineral composition (mg/kg dm) of 13 quinoa varieties grown under North-West European field conditions in 2017, 2018, and 2019 (*n* = 1), Table S8. Rotated structure matrix for principal components analysis with Varimax rotation, Table S9. Results of analysis of variance (model, Akaike information criteria, *p*-value).
