# Supplementary material for: Yield and Nutritional Characterization of Thirteen Quinoa (Chenopodium quinoa Willd.) Varieties Grown in North-West Europe—Part I
Source: Plants (Basel). 2021 Dec 7;10(12):2689. doi: 10.3390/plants10122689 (PMC8705647; doi:10.3390/plants10122689)
Supplement: Supplementary file 1 [file plants-10-02689-s001.zip › plants-1448994-supplementary.pdf]

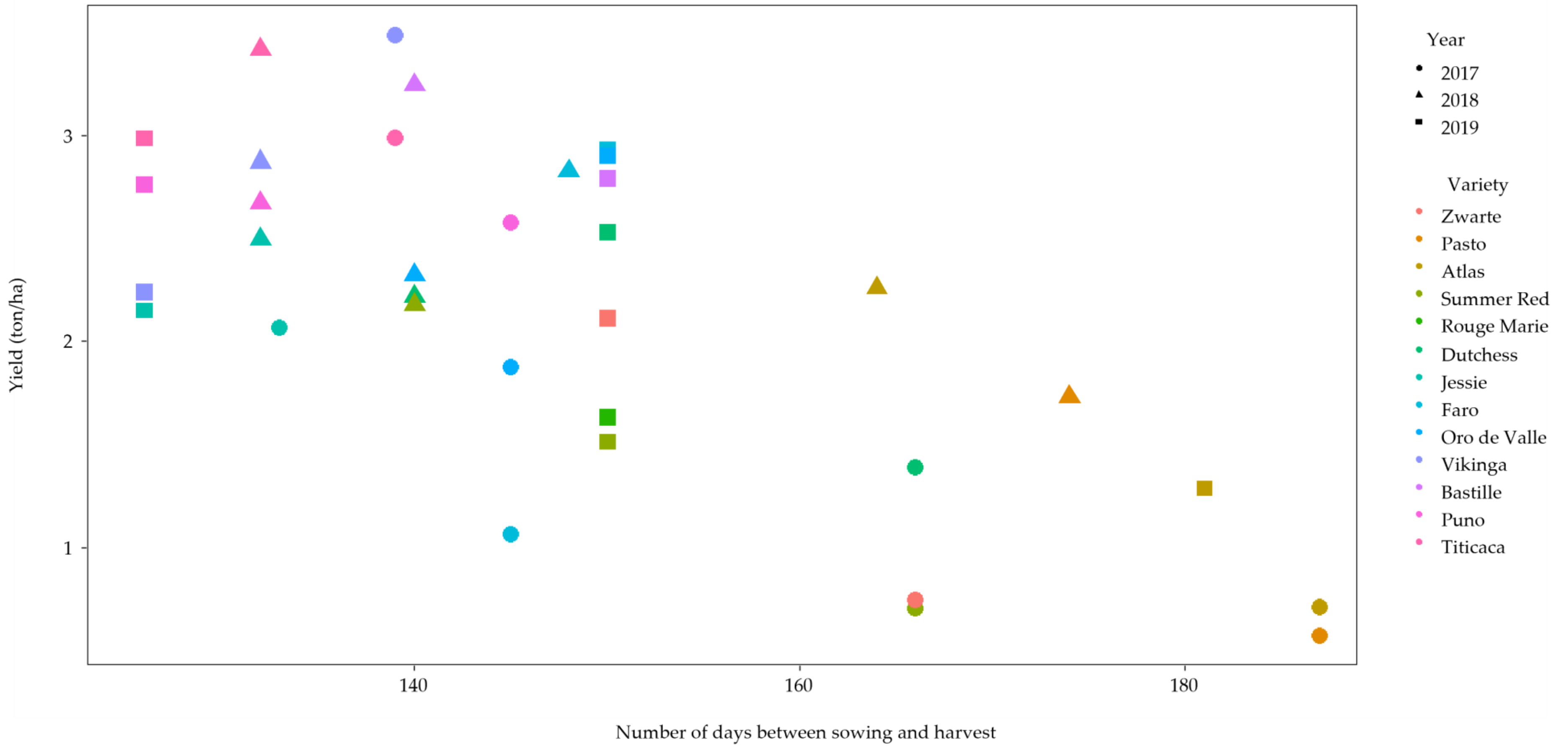

Figure S1. Correlation between yield and growing days (r = -0.705, p < 0.001) of thirteen quinoa varieties grown under North-West European field conditions in 2017, 2018 and 2019.

Table S1. Length-width ratio (LWR) and color parameters (L*a*b*) of thirteen quinoa varieties grown under North-West European field conditions in 2017, 2018 and 2019 ($n = 3$).

Year	Variety	LWR [1,2]	L *,1	a *,1	b *,1
2017	Atlas	1.10 ± 0.00[bcB]	64.76 ± 0.08 [fA]	3.57 ± 0.05 [bcA]	17.04 ± 0.01 [eB]
	Bastille	-	-	-	-
	Dutchess	1.09 ± 0.00 [aA]	63.78 ± 0.10 [eA]	4.59 ± 0.03 [fgB]	18.04 ± 0.03 [fB]
	Faro	1.10 ± 0.00 [bcB]	66.94 ± 0.42 [iB]	4.28 ± 0.07 [defB]	18.70 ± 0.12 [gB]
	Jessie	1.09 ± 0.00 [aA]	63.14 ± 0.51 [dA]	4.51 ± 0.03 [efgB]	18.87 ± 0.10 [gB]
	Oro de Valle	1.13 ± 0.00 [eC]	65.51 ± 0.09 [gC]	4.09 ± 0.06 [deA]	18.73 ± 0.13 [gB]
	Pasto	1.10 ± 0.00 [bcA]	60.55 ± 0.19 [cA]	3.98 ± 0.01 [cdA]	15.23 ± 0.07 [cA]
	Puno	1.11 ± 0.00 [cdC]	66.32 ± 0.06 [hB]	3.27 ± 0.01 [bA]	16.84 ± 0.01 [eA]
	Rouge Marie	-	-	-	-
	Summer Red	1.13 ± 0.00 [eB]	41.78 ± 0.18 [bA]	4.38 ± 0.78 [defgB]	2.21 ± 0.54 [bA]
	Titicaca	1.12 ± 0.00 [dB]	66.75 ± 0.15 [hiC]	3.20 ± 0.04 [bA]	16.40 ± 0.05 [dA]
	Vikinga	1.10 ± 0.00 [abA]	64.54 ± 0.36 [fB]	4.83 ± 0.05 [gA]	19.04 ± 0.04 [gC]
	Zwarte	1.14 ± 0.00 [fB]	40.81 ± 0.53 [aA]	1.91 ± 0.37 [aA]	1.11 ± 0.33 [aB]
2018	Atlas	1.09 ± 0.00 [aA]	65.65 ± 0.08 [fB]	3.82 ± 0.08 [aA]	15.80 ± 0.06 [bA]
	Bastille	1.12 ± 0.00 [eA]	67.69 ± 0.04 [hB]	4.15 ± 0.03 [aA]	19.06 ± 0.02 [fA]
	Dutchess	1.10 ± 0.00 [cdB]	63.83 ± 0.08 [cA]	4.14 ± 0.02 [aA]	16.98 ± 0.01 [cdA]
	Faro	1.1 ± 0.00 [bcdAB]	66.71 ± 0.02 [gB]	4.04 ± 0.02 [aAB]	18.38 ± 0.07 [eA]
	Jessie	1.09 ± 0.00 [aA]	66.29 ± 0.02 [gB]	4.07 ± 0.04 [aA]	18.26 ± 0.02 [eA]
	Oro de Valle	1.11 ± 0.00 [dB]	64.45 ± 0.06 [deB]	3.92 ± 0.02 [aA]	16.80 ± 0.03 [cdA]
	Pasto	1.10 ± 0.00 [bcdA]	62.48 ± 0.07 [bB]	4.83 ± 0.03 [bB]	17.17 ± 0.09 [dB]
	Puno	1.09 ± 0.00 [aA]	64.10 ± 0.10 [cdA]	3.72 ± 0.01 [aB]	16.60 ± 0.10 [cA]
	Rouge Marie	-	-	-	-
	Summer Red	1.09 ± 0.00 [abA]	43.99 ± 0.41 [aB]	3.88 ± 0.49 [aA]	2.66 ± 0.40 [aB]
	Titicaca	1.10 ± 0.00 [abcA]	64.87 ± 0.09 [eB]	3.84 ± 0.05 [aB]	16.70 ± 0.07 [cB]
	Vikinga	1.10 ± 0.00 [bcdAB]	62.43 ± 0.06 [bA]	4.83 ± 0.03 [bA]	18.15 ± 0.03 [eB]
	Zwarte	-	-	-	-
2019	Atlas	1.09 ± 0.00 [abcA]	64.56 ± 0.01 [fA]	4.21 ± 0.01 [bcB]	18.29 ± 0.02 [eC]
	Bastille	1.12 ± 0.00 [eA]	66.72 ± 0.02 [kA]	5.06 ± 0.01 [efB]	20.72 ± 0.01 [iB]
	Dutchess	1.10 ± 0.00 [bcdB]	64.65 ± 0.01 [fgB]	4.68 ± 0.01 [deB]	18.87 ± 0.01 [fC]
	Faro	1.10 ± 0.00 [bcdA]	65.50 ± 0.01 [hiA]	3.86 ± 0.01 [bA]	19.55 ± 0.01 [gC]

Jessie	1.10 ± 0.00 cdB	65.98 ± 0.01 ijB	4.82 ± 0.01 deB	19.71 ± 0.01 gC
Oro de Valle	1.09 ± 0.00 abcA	63.44 ± 0.01 eA	5.09 ± 0.01 efB	20.21 ± 0.01 hC
Pasto	-	-	-	-
Puno	1.10 ± 0.00 cdB	66.48 ± 0.01 jkB	4.05 ± 0.01 bC	18.86 ± 0.03 fB
Rouge Marie	1.09 ± 0.00 aA	45.69 ± 0.01 bA	6.56 ± 0.01 gA	5.48 ± 0.01 cA
Summer Red	1.09 ± 0.00 abA	47.12 ± 0.01 cC	4.56 ± 0.02 cdB	4.93 ± 0.01 bC
Titicaca	1.10 ± 0.00 cdA	60.15 ± 0.02 dA	5.42 ± 0.01 fC	18.14 ± 0.01 deC
Vikinga	1.10 ± 0.00 dB	65.11 ± 0.01 ghC	4.80 ± 0.01 deA	17.76 ± 0.01 dA
Zwarte	1.10 ± 0.00 dA	41.38 ± 0.01 aB	1.90 ± 0.01 aA	0.69 ± 0.01 aA

[1] Within years, average values followed by the same lowercase letter are not significantly different ($p > 0.05$). Capital letters compare the three years for the same variety, average values followed by the same letter are not significantly different ($p > 0.05$). [2] LWR: length-width ratio.

Table S2. Macronutrient composition (g/100 g dm) of thirteen quinoa varieties grown under North-West European field conditions in 2017, 2018 and 2019 (n = 3).

Year	Variety	Protein (g/100 g dm) [1]	Fat (g/100 g dm)[1]	Starch (g/100 g dm) [1]	Ash (g/100 g dm) [1]
2017	Atlas	16.6 ± 0.2 ^{fA}	6.41 ± 0.04 ^{cA}	58.1 ± 0.6 ^{dB}	3.46 ± 0.02 ^{gC}
	Bastille	-	-	-	-
	Dutchess	13.8 ± 0.0 ^{cA}	7.03 ± 0.07 ^{eA}	59.6 ± 0.5 ^{eB}	3.35 ± 0.01 ^{fB}
	Faro	14.9 ± 0.3 ^{dB}	7.33 ± 0.09 ^{fA}	53.4 ± 0.7 ^{aB}	3.32 ± 0.02 ^{efB}
	Jessie	16.0 ± 0.2 ^{eB}	6.70 ± 0.04 ^{dA}	54.9 ± 0.7 ^{bA}	3.12 ± 0.02 ^{dC}
	Oro de Valle	12.1 ± 0.2 ^{aA}	6.18 ± 0.03 ^{cA}	67.2 ± 1.2 ^{gC}	2.86 ± 0.03 ^{bA}
	Pasto	15.5 ± 0.3 ^{eA}	5.42 ± 0.05 ^{aA}	56.5 ± 0.5 ^{cA}	3.47 ± 0.02 ^{gA}
	Puno	13.9 ± 0.2 ^{cB}	5.65 ± 0.02 ^{abA}	61.7 ± 0.4 ^{fB}	2.37 ± 0.02 ^{aA}
	Rouge Marie	-	-	-	-
	Summer Red	13.9 ± 0.2 ^{cB}	8.54 ± 0.08 ^{iC}	58.0 ± 0.2 ^{dC}	3.29 ± 0.01 ^{eB}
	Titicaca	14.8 ± 0.1 ^{dB}	7.61 ± 0.08 ^{gB}	60.7 ± 0.8 ^{fA}	2.84 ± 0.01 ^{bAB}
	Vikinga	14.9 ± 0.4 ^{dB}	8.04 ± 0.08 ^{hB}	57.8 ± 0.7 ^{dA}	3.02 ± 0.03 ^{cB}
	Zwarte	13.0 ± 0.3 ^{bA}	5.70 ± 0.03 ^{bA}	55.2 ± 0.7 ^{bA}	3.16 ± 0.03 ^{dA}
2018	Atlas	16.4 ± 0.1 ^{fA}	7.78 ± 0.03 ^{eC}	57.5 ± 0.5 ^{aB}	3.30 ± 0.02 ^{cB}
	Bastille	13.1 ± 0.2 ^{bA}	7.34 ± 0.01 ^{dA}	64.0 ± 0.3 ^{eB}	3.09 ± 0.03 ^{bB}
	Dutchess	14.4 ± 0.2 ^{eB}	7.75 ± 0.04 ^{eB}	61.4 ± 0.4 ^{cC}	3.32 ± 0.02 ^{cB}
	Faro	13.2 ± 0.2 ^{bA}	8.09 ± 0.08 ^{fC}	58.2 ± 0.4 ^{abC}	3.07 ± 0.01 ^{bA}
	Jessie	13.7 ± 0.1 ^{cdA}	7.93 ± 0.05 ^{efB}	59.1 ± 0.4 ^{bC}	3.08 ± 0.01 ^{bB}
	Oro de Valle	12.2 ± 0.1 ^{aA}	7.73 ± 0.06 ^{eC}	62.5 ± 0.6 ^{dB}	3.08 ± 0.01 ^{bC}
	Pasto	16.0 ± 0.2 ^{fB}	6.37 ± 0.04 ^{aB}	59.0 ± 0.6 ^{bB}	3.60 ± 0.02 ^{dB}
	Puno	12.5 ± 0.2 ^{aA}	6.67 ± 0.03 ^{bC}	63.7 ± 0.3 ^{eC}	2.90 ± 0.02 ^{aC}
	Rouge Marie	-	-	-	-
	Summer Red	13.3 ± 0.1 ^{bcA}	8.18 ± 0.01 ^{fB}	57.3 ± 0.3 ^{aB}	3.27 ± 0.03 ^{cB}
	Titicaca	13.2 ± 0.1^{bA}	7.08 ± 0.03 ^{cA}	63.9 ± 0.7 ^{eB}	2.88 ± 0.01 ^{aB}
	Vikinga	13.8 ± 0.4 ^{dA}	8.50 ± 0.09 ^{gC}	63.6 ± 0.2 ^{eC}	3.08 ± 0.03 ^{bC}
	Zwarte	-	-	-	-
2019	Atlas	18.5 ± 0.0 ^{fgB}	7.20 ± 0.14 ^{dB}	54.5 ± 0.4 ^{cA}	3.03 ± 0.02 ^{cdA}
	Bastille	16.6 ± 0.0 ^{bcB}	7.36 ± 0.19 ^{deA}	52.3 ± 0.5 ^{bA}	3.01 ± 0.03 ^{cdA}
	Dutchess	18.4 ± 0.1 ^{fgC}	7.17 ± 0.04 ^{dA}	54.8 ± 0.2 ^{cdA}	3.21 ± 0.01 ^{fA}

Faro	17.6 ± 0.1 ^{dC}	7.61 ± 0.12 ^{efB}	50.5 ± 0.4 ^{aA}	3.09 ± 0.01 ^{deA}
Jessie	18.8 ± 0.0 ^{gC}	6.70 ± 0.24 ^{cA}	55.6 ± 1.3 ^{deB}	2.84 ± 0.03 ^{aC}
Oro de Valle	16.2 ± 0.0 ^{abB}	7.22 ± 0.07 ^{dB}	54.7 ± 0.8 ^{cdA}	2.91 ± 0.02 ^{bB}
Pasto	-	-	-	-
Puno	17.0 ± 0.1 ^{cC}	5.88 ± 0.24 ^{aB}	59.4 ± 1.1 ^{fA}	2.83 ± 0.01 ^{aB}
Rouge Marie	17.8 ± 0.1 ^{deA}	7.65 ± 0.08 ^{fA}	53.9 ± 0.2 ^{cA}	3.10 ± 0.02 ^{eA}
Summer Red	18.1 ± 0.2 ^{efC}	7.75 ± 0.09 ^{fA}	54.5 ± 0.4 ^{cA}	3.20 ± 0.01 ^{fA}
Titicaca	16.0 ± 0.1 ^{aC}	7.25 ± 0.03 ^{dA}	72.5 ± 0.9 ^{hC}	2.83 ± 0.02 ^{aA}
Vikinga	17.0 ± 0.1 ^{cC}	7.71 ± 0.04 ^{fA}	60.8 ± 0.5 ^{gB}	2.82 ± 0.02 ^{aA}
Zwarte	16.0 ± 0.1 ^{aB}	6.36 ± 0.08 ^{bB}	56.2 ± 0.2 ^{eB}	3.29 ± 0.04 ^{gB}

[1] Within years, average values followed by the same lowercase letter are not significantly different ($p > 0.05$). Capital letters compare the three years for the same variety, average values followed by the same letter are not significantly different ($p > 0.05$).

Table S3. Non- and semi-essential amino acid composition (mg/g protein) of thirteen quinoa varieties grown under North-West European field conditions in 2017, 2018 and 2019 (*n* = 1).

Year	Variety	Amino Acid (mg/g protein)									
		Tyr	*Ser*	*Ala*	*Pro*	*THP*	*Gly*	*Glu*	*Asp*	*Cys*	*Arg*
2017	Atlas	41	37	43	33	24	64	153	117	20	100
	Dutchess	37	35	52	33	22	50	146	127	21	89
	Faro	37	38	46	31	22	50	148	113	24	98
	Jessie	31	38	38	28	19	45	145	117	19	88
	Oro de Valle	40	38	50	32	25	50	151	138	22	92
	Pasto	39	41	50	35	21	56	154	124	21	97
	Puno	35	37	43	30	19	48	155	116	22	100
	Summer Red	38	39	43	33	22	48	146	146	20	94
	Titicaca	36	40	43	31	19	53	156	120	24	98
	Vikinga	35	42	47	33	19	53	158	114	23	101
	Zwarte	36	39	50	34	23	44	137	132	21	87
2018	Atlas	37	41	41	29	18	52	137	77	14	93
	Bastille	33	33	44	27	15	50	146	81	16	76
	Dutchess	41	37	52	30	16	58	183	94	20	100
	Faro	38	31	48	31	17	62	160	104	21	101
	Jessie	39	38	63	33	16	61	161	103	20	94
	Oro de Valle	36	36	47	32	17	58	144	97	19	78
	Pasto	36	35	47	29	16	54	173	94	19	99
	Puno	41	39	57	32	17	64	179	107	23	103
	Summer Red	38	34	50	31	18	59	166	96	18	96
	Titicaca	39	35	53	31	18	61	165	103	21	98
	Vikinga	33	35	49	29	14	57	153	98	19	93
2019	Atlas	38	35	42	27	21	53	131	98	17	86
	Bastille	36	37	42	31	21	56	142	106	17	88
	Dutchess	41	41	48	32	24	65	154	109	22	100
	Faro	37	36	39	27	23	53	132	103	21	92

Jessie	37	40	45	30	21	57	137	104	16	89
Oro de Valle	46	42	55	49	25	74	177	179	22	117
Puno	42	44	43	40	21	65	165	135	20	116
Rouge Marie	38	40	46	35	22	62	136	121	15	100
Summer Red	37	42	39	34	21	57	134	112	16	98
Titicaca	40	36	49	40	23	69	152	125	20	101
Vikinga	41	36	44	36	23	58	149	124	20	103
Zwarte	35	42	45	33	19	54	151	114	24	99

Table S4. Essential amino acid composition (mg/g protein) of thirteen quinoa varieties grown under North-West European field conditions in 2017, 2018 and 2019 (n = 1).

Year	**Variety**	**Amino Acid (mg/g protein)**									
		Phe	*Leu*	*Met*	*Ile*	*Val*	*Thr*	*His*	*Lys*	*Trp*	*essential*
2017	Atlas	47	86	24	52	72	38	34	61	16	429
	Bastille	-	-	-	-	-	-	-	-	-	-
	Dutchess	38	78	23	42	67	44	34	57	17	400
	Faro	38	74	24	39	63	45	35	58	11	386
	Jessie	35	65	21	35	54	40	31	51	13	344
	Oro de Valle	40	77	26	41	67	46	35	62	15	408
	Pasto	39	79	24	44	70	51	36	63	-	-
	Puno	38	73	22	39	59	44	34	55	16	380
	Rouge Marie	-	-	-	-	-	-	-	-	-	-
	Summer Red	36	75	22	39	63	43	32	52	18	380
	Titicaca	38	73	23	39	62	45	36	58	17	390
	Vikinga	37	68	23	38	60	43	36	58	13	375
	Zwarte	37	73	23	39	62	44	31	55	13	378
2018	Atlas	46	67	17	37	42	33	27	49	19	338
	Bastille	43	62	23	39	47	35	20	52	23	344
	Dutchess	49	70	26	43	53	38	23	58	19	379
	Faro	50	69	32	41	51	39	23	60	19	383
	Jessie	50	75	31	46	60	44	22	64	20	413
	Oro de Valle	47	65	27	38	46	38	22	56	21	360
	Pasto	45	62	25	39	45	35	22	54	16	342
	Puno	50	75	32	45	56	41	24	63	20	407
	Rouge Marie	-	-	-	-	-	-	-	-	-	-
	Summer Red	46	64	32	40	47	39	24	56	21	370
	Titicaca	51	72	26	44	53	39	24	58	24	391

	Vikinga	44	64	26	37	48	35	21	55	23	352
	Zwarte	-	-	-	-	-	-	-	-	-	-
2019	Atlas	40	76	20	47	60	37	30	51	16	376
	Bastille	42	80	20	45	59	37	29	50	15	378
	Dutchess	43	80	22	46	64	40	32	55	14	396
	Faro	42	79	20	44	62	37	32	52	14	382
	Jessie	41	76	21	44	67	37	29	51	14	380
	Oro de Valle	49	98	29	51	73	35	33	58	17	443
	Pasto	-	-	-	-	-	-	-	-	-	-
	Puno	46	86	24	53	67	38	36	56	14	419
	Rouge Marie	41	82	21	48	64	36	30	46	16	382
	Summer Red	39	74	21	44	61	35	30	48	14	366
	Titicaca	45	77	22	47	64	35	32	53	18	393
	Vikinga	44	80	22	47	66	34	32	52	16	393
	Zwarte	36	72	22	39	65	43	34	60	16	387

Table S5. Fatty acid composition (% fatty acid methyl ester) of thirteen quinoa varieties grown under North-West European field conditions in 2017, 2018 and 2019 (n = 1).

Year	Variety	Fatty Acid (% FAME) [1]											Ω6/Ω3
		C16:0	*C18:0*	*C18:1c*	*C18:2 n-6*	*C18:3 n-3*	*C20:1*	*C22:1*	*C22:2*	*SFA*	*MUFA*	*PUFA*	
2017	Atlas	9.0	0.9	20.7	54.9	5.4	1.0	1.3	4.6	10.9	23.5	65.4	10.1
	Bastille	-	-	-	-	-	-	-	-				
	Dutchess	8.7	0.7	20.6	53.3	5.5	0.9	1.0	7.5	10.3	23.0	66.6	9.8
	Faro	9.0	0.7	19.4	56.7	6.7	1.1	1.3	2.9	10.6	22.2	67.1	8.5
	Jessie	9.2	0.5	18.9	56.8	6.4	1.0	1.4	3.9	10.7	21.6	67.4	8.9
	Oro de Valle	9.2	0.5	20.8	55.4	6.8	1.1	1.3	2.7	10.9	23.5	65.5	8.2
	Pasto	9.4	1.0	19.5	54.2	6.5	1.1	1.2	4.3	11.7	22.5	65.7	8.3
	Puno	9.1	0.5	16.9	58.8	7.1	1.1	1.2	3.2	10.8	19.5	69.6	8.2
	Rouge Marie	-	-	-	-	-	-	-	-				
	Summer Red	8.7	0.6	19.3	54.9	5.0	1.0	1.3	7.2	10.2	22.1	67.6	10.9
	Titicaca	9.9	0.7	19.0	57.2	5.8	1.1	1.2	3.2	11.9	21.5	66.4	10.0
	Vikinga	9.9	0.5	21.0	54.9	5.9	1.1	1.3	3.4	11.5	23.8	64.4	9.4
	Zwarte	9.3	0.8	19.3	56.1	7.1	1.1	1.1	3.2	11.2	21.9	66.8	8.0
2018	Atlas	9.9	0.7	21.3	55.4	5.1	1.3	1.4	2.5	12.0	24.4	63.4	10.9
	Bastille	9.5	0.6	20.4	54.5	7.6	1.2	1.2	2.5	11.5	23.3	65.1	7.2
	Dutchess	9.9	0.6	21.4	55.2	5.2	1.2	1.4	2.7	11.8	24.5	63.5	10.6
	Faro	8.8	0.6	20.9	58.0	5.3	1.2	1.3	1.6	10.7	23.9	65.3	11.0
	Jessie	9.1	0.5	20.1	57.2	5.5	1.1	1.4	3.2	10.7	23.1	66.0	10.4
	Oro de Valle	8.8	0.5	20.7	57.4	6.8	1.2	1.1	1.4	10.6	23.4	65.9	8.5
	Pasto	9.8	0.6	19.2	55.4	7.0	1.3	1.5	2.8	11.6	22.7	65.6	7.9
	Puno	9.0	0.5	18.1	58.5	6.8	1.1	1.3	2.5	10.6	21.3	68.0	8.6
	Rouge Marie	-	-	-	-	-	-	-	-				
	Summer Red	9.9	0.7	21.6	56.2	4.7	1.3	1.4	2.1	11.8	24.7	63.3	12.0

	Titicaca	11.4	0.7	20.6	55.9	4.8	1.2	1.1	2.3	13.4	23.2	63.3	11.6
	Vikinga	11.3	0.7	22.3	54.0	5.0	1.2	1.3	2.2	13.3	25.1	61.4	10.8
	Zwarte	-	-	-	-	-	-	-	-				
2019	Atlas	9.1	0.6	20.7	55.7	5.8	1.3	1.4	2.8	11.1	23.8	64.9	9.6
	Bastille	9.3	0.6	19.4	54.7	8.1	1.2	1.2	3.0	11.1	22.3	66.4	6.7
	Dutchess	9.6	0.6	19.9	55.4	6.1	1.1	1.4	3.3	11.5	22.9	65.4	9.0
	Faro	8.8	0.6	19.3	57.5	6.4	1.1	1.2	2.9	10.6	22.0	67.3	9.0
	Jessie	9.2	0.4	17.3	57.8	7.0	1.2	1.5	3.1	10.8	20.3	68.7	8.3
	Oro de Valle	9.4	0.5	20.2	56.3	6.5	1.2	1.3	2.5	11.1	23.0	65.7	8.7
	Pasto	-	-	-	-	-	-	-	-				
	Puno	9.5	0.5	15.5	59.8	7.7	1.0	1.3	2.5	11.1	18.1	70.6	7.8
	Rouge Marie	10.0	0.6	19.7	56.4	4.8	1.2	1.6	3.5	11.8	22.9	65.1	11.8
	Summer Red	10.0	0.6	19.7	56.4	4.8	1.2	1.5	3.4	11.8	23.0	65.1	11.8
	Titicaca	9.6	0.5	19.7	57.0	6.0	1.2	1.2	2.8	11.3	22.3	66.2	9.5
	Vikinga	10.0	0.5	20.2	55.3	6.5	1.1	1.4	2.7	11.8	23.1	64.9	8.5
	Zwarte	9.7	0.6	18.4	57.0	7.1	1.0	1.2	2.6	11.6	21.1	67.2	8.1

[1] FAME: fatty acid methyl esters, SFA: saturated fatty acids, MUFA: monounsaturated fatty acids, PUFA: polyunsaturated fatty acids.

Table S6. Triacylglycerol composition (area %) of thirteen quinoa varieties grown under North-West European field conditions in 2017, 2018 and 2019 (n = 1).

Year	Variety	Triacylglycerol (area %)									
		LnLnL	*LLLn*	*LLL*	*OLLn*	*LLnP*	*LLO*	*LLP*	*OOL*	*OLP*	*PPL*
2017	Atlas	0.49	6.45	24.74	3.61	1.34	27.94	9.99	7.80	5.84	2.43
	Dutchess	0.63	5.04	24.53	2.79	1.00	32.24	9.45	8.12	5.80	2.30
	Faro	1.68	8.21	17.19	5.85	3.54	20.45	11.08	8.00	7.96	3.35
	Jessie	0.93	6.79	18.61	4.46	2.69	21.57	12.53	8.44	8.54	3.87
	Oro de Valle	0.66	7.61	23.29	4.35	1.66	26.03	10.44	8.18	6.62	2.40
	Pasto	0.76	7.98	22.62	4.52	2.00	26.00	10.27	6.75	5.88	2.56
	Puno	1.20	9.10	24.03	4.38	2.35	22.29	12.61	6.15	6.53	3.03
	Summer Red	0.48	5.62	26.20	2.92	1.26	29.09	11.03	6.79	5.78	2.54
	Titicaca	0.86	6.89	21.75	4.18	1.89	23.49	13.37	7.93	8.08	3.06
	Vikinga	0.63	7.09	20.91	4.59	2.11	25.29	10.70	7.40	6.54	3.00
	Zwarte	0.84	8.53	21.26	4.83	2.37	23.38	11.06	7.35	7.08	2.75
2018	Atlas	0.54	5.23	17.75	3.54	2.02	22.13	12.14	9.26	9.23	4.42
	Bastille	1.09	7.04	16.62	5.23	3.11	20.78	12.17	8.42	8.86	3.67
	Dutchess	0.51	5.19	17.20	3.82	2.01	22.13	12.54	9.44	9.85	4.29
	Faro	0.53	5.88	21.44	3.73	1.57	23.37	12.08	9.24	8.14	3.35
	Jessie	0.48	6.33	19.89	3.85	1.97	22.64	12.15	8.34	8.80	3.98
	Oro de Valle	0.82	7.55	20.34	4.37	2.15	22.05	11.75	8.75	7.80	3.37
	Pasto	0.92	6.95	16.96	4.62	3.18	20.57	12.67	7.64	8.51	4.61
	Puno	0.88	8.09	22.08	4.37	2.39	21.78	13.01	6.86	7.61	3.54
	Summer Red	0.45	5.05	17.83	3.58	1.92	22.74	12.49	9.29	9.66	4.26
	Titicaca	0.42	5.66	20.16	3.69	1.96	22.64	13.03	8.91	9.09	3.53
	Vikinga	0.40	5.15	17.88	3.92	1.89	23.15	12.62	10.22	9.67	3.54
2019	Atlas	0.59	6.54	18.38	4.34	2.32	21.94	11.75	9.05	8.14	3.89
	Bastille	1.22	7.95	19.30	5.28	2.74	23.30	11.79	7.85	7.82	2.80

Dutchess	0.99	6.77	17.38	4.51	2.82	20.65	12.60	8.61	8.84	4.17
Faro	0.59	7.25	21.49	4.51	2.00	22.87	11.56	8.25	7.24	3.03
Jessie	0.84	8.61	22.18	4.67	2.70	21.43	12.56	6.26	7.13	3.69
Oro de Valle	1.60	9.61	22.53	4.39	3.24	19.14	13.87	5.60	6.77	3.67
Puno	1.51	9.56	23.72	4.26	3.10	19.50	13.93	5.35	6.66	3.43
Rouge Marie	0.57	5.20	18.77	3.42	2.17	22.37	13.29	8.61	9.35	4.51
Summer Red	0.53	5.21	18.90	3.39	2.11	22.66	13.57	8.60	9.37	4.44
Titicaca	0.89	6.61	19.94	4.27	2.42	22.15	13.07	8.29	8.61	3.49
Vikinga	0.94	6.53	18.32	4.56	2.68	21.54	12.92	8.82	9.02	3.68
Zwarte	1.18	8.05	19.46	4.63	3.07	20.48	12.98	7.44	8.18	3.63

Table S7. Mineral composition (mg/kg dm) of thirteen quinoa varieties grown under North-West European field conditions in 2017, 2018 and 2019 (n = 1).

Year	Variety	Mineral (mg/kg dm)									
		B	*Ca*	*Cu*	*Fe*	*K*	*Mg*	*Mn*	*Na*	*P*	*Zn*
2017	Atlas	12	551	10	74	12478	2308	20	118	5775	59
	Bastille	-	-	-	-	-	-	-	-	-	-
	Dutchess	12	655	10	65	13421	2211	19	297	5016	42
	Faro	13	646	8	64	11216	2476	25	101	5674	46
	Jessie	12	535	8	64	10520	2377	22	82	5588	45
	Oro de Valle	11	583	6	127	10580	1866	21	91	4141	49
	Pasto	13	748	19	231	12259	2671	32	165	5188	68
	Puno	10	540	14	63	8790	2072	19	226	4266	45
	Rouge Marie	-	-	-	-	-	-	-	-	-	-
	Summer Red	15	500	7	53	12934	2244	27	127	4919	46
	Titicaca	11	413	7	333	9261	2028	21	106	4680	49
	Vikinga	13	654	8	63	10481	2192	24	82	4573	51
	Zwarte	14	582	11	50	14053	2025	28	241	4515	39
2018	Atlas	13	649	179	80	10359	2481	31	107	6053	57
	Bastille	9	597	3	723	11072	2263	32	95	4782	109
	Dutchess	11	623	15	187	11023	2447	28	142	6019	45
	Faro	14	669	10	475	9664	2682	45	67	5619	45
	Jessie	10	489	10	80	9748	2420	27	87	6156	45
	Oro de Valle	13	617	8	64	10884	2345	35	78	5409	43
	Pasto	9	806	12	91	12819	2742	46	78	5373	67
	Puno	10	532	8	72	9440	2368	28	91	4981	43
	Rouge Marie	-	-	-	-	-	-	-	-	-	-
	Summer Red	11	399	8	59	12120	2179	30	90	5538	40
	Titicaca	11	437	2	136	9377	2322	21	75	5208	47
	Vikinga	12	491	9	86	9719	2314	27	81	5025	44
	Zwarte	-	-	-	-	-	-	-	-	-	-

2019	Atlas	16	640	10	78	10328	2109	22	65	5321	64
	Bastille	15	676	9	61	11722	2068	22	52	4202	43
	Dutchess	15	782	9	77	11722	2267	23	42	4998	47
	Faro	15	742	8	71	11024	2300	38	50	4678	54
	Jessie	15	497	9	75	10398	2143	24	49	4854	50
	Oro de Valle	18	697	8	65	11623	2059	31	55	4240	51
	Pasto	-	-	-	-	-	-	-	-	-	-
	Puno	14	657	5	64	11301	2052	25	55	3720	45
	Rouge Marie	15	515	7	64	12025	2000	22	57	4766	44
	Summer Red	16	524	8	61	12595	2048	22	48	4852	44
	Titicaca	13	500	5	58	10968	2106	22	58	4124	38
	Vikinga	18	665	7	71	10303	2102	24	51	4291	47
	Zwarte	16	734	7	59	13380	2074	28	46	4516	42

Table S8. Rotated structure matrix for principal components analysis with Varimax rotation.

**Variables** [1]	**PC1** [2,3]	**PC2** [2,3]	**PC3** [2,3]
length	**0.976**	-0.050	-0.103
width	**0.968**	-0.113	-0.082
TSW	**0.897**	-0.105	-0.015
protein	-0.134	**-0.894**	0.035
test weight	-0.186	**0.785**	0.120
starch	-0.184	**0.740**	0.155
yield	-0.326	0.228	**0.820**
fat	0.281	-0.114	**0.704**
ash	0.317	-0.299	**-0.520**

[1] TSW: thousand seed weight [2] PC: principal component [3] Major loadings for each variable are bolded.

Table S9. Results of analysis of variance (model, Akaike information criteria, *p* value).

**Variable** [1]	**Model**	**AIC** [2]	**ANOVA** [2]
seed length	Variety × Year	-614.7	< 0.001
seed width	Variety × Year	-615.5	< 0.001
TSW	Variety × Year + Replicate	-183.6	< 0.001
test weight	Variety × Year + Replicate	56.2	< 0.001
saponins	Variety × Year + Replicate	158.3	< 0.001
LWR	Variety × Year	-860.0	< 0.001
L* seed	Variety × Year	-25.9	< 0.001
a* seed	Variety × Year	-46.8	< 0.001
b* seed	Variety × Year	-85.5	< 0.001
protein	Variety × Year + Replicate	62.8	< 0.001
fat	Variety × Year	-171.5	< 0.001
starch	Variety × Year	567.8	< 0.001
ash	Variety × Year	-466.8	< 0.001

[1] TSW: thousand seed weight; LWR: length-width ratio [2] AIC: Akaike information criteria, ANOVA: analysis of variance.
